# Supplementary material for: A Nutrition Counseling Curriculum to Address Cardiovascular Risk Reduction for Internal Medicine Residents
Source: MedEdPORTAL. 2020 Nov 11;16:11027. doi: 10.15766/mep_2374-8265.11027 (PMC7666832; doi:10.15766/mep_2374-8265.11027)
Supplement: Supplementary file 1 — Session 1 Preceptor Handout.docxSession 1 Resident Handout.docxSession 2 Preceptor Handout.docxSession 2 Resident Handout.docxTake-Home Handout.docxPre-and Postsurvey.docx [file mep_2374-8265.11027-s001.zip › A. Session 1 Preceptor Handout.docx]

NUTRITION COUNSELING TO REDUCE CARDIOVASCULAR RISK- SESSION 1

*Please allow 5-10 minutes at the beginning of the pre-clinic conference for residents to complete the pre-curriculum survey.

OBJECTIVES

1. Improve resident attitudes regarding the role of the primary care provider in nutrition counseling for patients with hypertension, hyperlipidemia, overweight and obesity, and cardiovascular disease.
2. Identify waist circumference as a risk factor for cardiovascular disease.
3. List evidence-based dietary recommendations for the management of overweight and obesity, HTN, HLD, and CVD.
4. Ms. Turner is a 45 yo F with BMI 29. She comes in for routine follow up. Her waist circumference at today’s visit is 37 in. Her blood pressure is 120/70.
   1. What are her risk factors for cardiovascular disease?
      1. **Overweight**. Overweight is classified as body mass index (BMI) between 25-29.9 kg/m^2^. It is a harbinger for obesity and its adverse consequences. BMI has a J-shaped association with cardiovascular disease and all-cause mortality, with the lowest risk occurring at 21-25 kg/m^2^.^1^ This means that as BMI increases above 25 kg/m^2^, risk for these conditions increases.
      2. **Increased waist circumference**. In patients with BMI between 25-35 kg/m^2^, increased waist circumference is independently associated with increased risk for type 2 diabetes, hyperlipidemia, hypertension, and cardiovascular disease.^2^ Waist circumference provides an estimate of increased abdominal fat even in the absence of changes in BMI, which is important because visceral adipose tissue (or abdominal fat tissue) is closely linked with metabolic dysfunction and insulin resistance.^2^


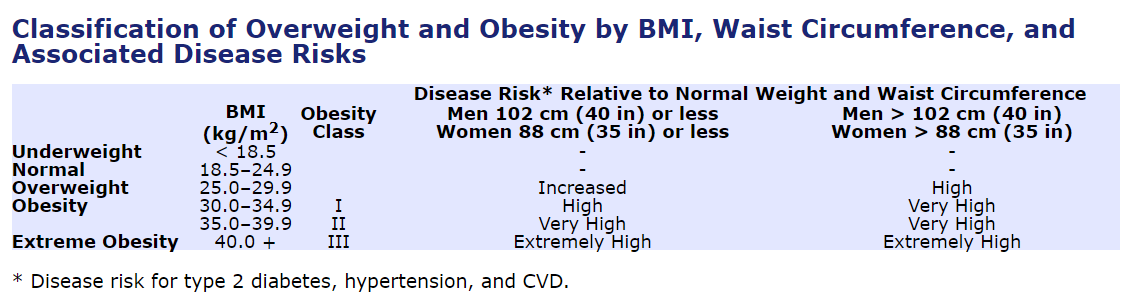


Image by National Heart, Lung, and Blood Institute; retrieved from <https://www.nhlbi.nih.gov/health/educational/lose_wt/BMI/bmi_dis.htm>, on 12/1/2018. Image is in the public domain.

- 1. How do you measure waist circumference? How often should you measure waist circumference?
     1. Waist circumference should be measured at least annually. You can ask your medical assistant to place a tape measure in the room for patients in whom you plan to measure waist circumference. Measure at the uppermost border of the iliac crest.
  2. You ask her how she feels about her weight and she replies that she wants to lose weight but isn’t sure where to start. How would you counsel this patient to help her lose weight?
     1. **2013 AHA Guidelines for Obesity Management: Achieve a negative calorie balance via exercise and reduced caloric intake** (Grade A, Class I, Level of Evidence A)
        1. No one diet plan (ie. low carbohydrate vs low fat) is best – individualize to disease states, patient preferences, and cultural preferences.
        2. General goals for caloric intake: 1200-1500 kcal/day for women and 1500-1800 kcal/day for men. An easier way to incorporate measuring calories may be to simply recommend trying to achieve a 500-750 kcal/day energy deficit.
        3. Physical activity- 150-300 min/week moderate intensity exercise or 75-150 min/week of vigorous intensity with at least 2 sessions/week of resistance/muscle-strengthening activity.
           1. Exercise is the best predictor of long-term weight loss and weight maintenance. It has also been shown to redistribute abdominal adiposity. Even modest increases in moderate-to-vigorous physical activity is beneficial.
     2. **Realistic goals to offer patients: 10% weight loss over 6 months (1-2 lb/week)**
        1. Choose 1-2 goals at a time
           1. Reduce sedentary activities
           2. Increase physical activity
           3. Reduce portion sizes of meals and snacks
           4. Limit or eliminate caloric beverages and choose water, unsweetened tea, or coffee
     3. Refer to comprehensive lifestyle program. The most effective interventions are comprehensive, in person, and high frequency/intensity.
  3. You see Ms. Turner for follow up 6 months later. She has since lost 5 lbs and her waist circumference is 35 in. You congratulate her on her success. She thanks you and states that although she has made some dietary changes, she is still worried about her diet. She says, “I mostly drink just water now instead of sweet tea, but is there anything else I should or should not be eating? Heart disease runs in my family and I’m worried about having a heart attack one day.”
     1. Have you provided nutrition counseling to your patients? What barriers have you come across that prevent you from routinely counseling about diet?
     2. What is the benefit of nutrition counseling for cardiovascular risk? What is the role of the PCP in these situations?
        1. **Even modest dietary changes and weight loss can lead to significant improvement in lipid profiles, blood pressure, glycemic control, and BMI/waist circumference**. The role of nutrition is central to these disease states, whether or not a patient is on medications for these diseases.
        2. **Suboptimal intake of 10 dietary factors was associated with 45% of cardiometabolic deaths** (heart disease, stroke, or T2DM) in 2012, from meta analyses of prospective cohorts and RCTs^3^. Specifically, these include excess sodium intake, insufficient intake of nuts/seeds, high intake of processed meats, and low intake of omega-3 fats.
        3. The PCP is the central player in the management of chronic diseases and nutrition modification plays a central role in that management, thus the PCP should be routinely addressing adherence to diet with their patients, whether or not they are on medications.
     3. What advice would you provide Ms. Turner in response to her question?
        1. **A heart healthy diet promotes consumption of whole grains, vegetables, fruit, legumes, nuts and seeds. It limits intake of meat and refined and processed foods.** This allows for intake of fiber, phytochemicals, mono and poly unsaturated fatty acids, and antioxidants. It also decreases intake of added sugars, sodium, and saturated and trans fats. We will explore why these changes are important throughout these two sessions.
           1. In large prospective cohort studies of patients without cardiovascular disease, cancer, or diabetes at baseline who were followed for 8-14 years (Nurses Health Study and Health Professionals Follow up Study), each daily serving increase in fruits or vegetables was associated with a 4% decreased risk for coronary heart disease. Green leafy vegetables had the largest effect: 23% decreased risk^4^.
           2. In the same cohort studies, 5 or more servings of nuts per week was associated with a 14% decreased risk for cardiovascular disease and 20% decreased risk for coronary heart disease^5^.
           3. In a meta-analysis of seven prospective cohort studies, an average of 2.5 servings of whole grains daily was associated with a 21% lower risk of CVD events^6^.
        2. **Two dietary patterns that are heart healthy are the Mediterranean and Plant Based Diets.**
           1. **Mediterranean eating pattern:**

**Choose whole grains, fruits and vegetables**

**Protein sources: Emphasize seafood, legumes (beans and peas), nuts/seeds. Moderate intake of poultry, eggs.**

**Olive oil as predominant cooking oil**

**Less emphasis on dairy compared to a standard US diet**

**Limit intake of red meat, added sugars**

- - - - 1. **Plant-based eating pattern: similar to Mediterranean diet.**

**Differences: limited or no intake of eggs and dairy; no meat, poultry, or seafood.**

- - - 1. Evidence for the Mediterranean diet
         1. In the PREDIMED trial, a primary prevention RCT of over 7400 patients in Spain, the Mediterranean diet was shown to decrease rates of MI, stroke, or CV death by 28-30% at 5 years^7^.
         2. In the Lyon Diet Heart Study, an RCT of patients with CV disease, the Mediterranean diet was shown to reduce risk of cardiac death, MI, stroke at 4 years by 47-72%^8^.
      2. Evidence for the plant-based diet:
         1. A meta-analysis of prospective cohort studies examining vegetarian and vegan diets reported a 25% reduced risk of incidence and mortality from ischemic heart disease^9^.
         2. Data from Nurses’ Health Study and Health Professionals Follow up Study showed that animal protein intake was associated with higher CV mortality, and plant protein was associated with lower all cause and CV mortality when participants had at least 1 unhealthy lifestyle factor. Substitution of 3% of energy from animal protein for plant protein was associated with lower all-cause mortality. This association was greatest for processed red meat: HR 0.66^10^.
         3. Processed meat has been declared carcinogenic by the WHO.

1. Mr. Smith is a 52 yo M with HTN and obesity who has a BMI of 36. His BP is 150/90 at today’s visit confirmed on manual recheck. He eats 3 meals/day and favors snack foods like chips and dip. He eats out 2-3 times/week at buffet style restaurants. He has 1 drink with hard liquor before dinner and 2 beers daily. He walks a block to work daily.
   1. What are targets for lifestyle intervention you could work on with this patient? What lifestyle change(s) would provide the greatest reduction in his blood pressure?
      1. 2013 AHA guidelines for lifestyle management to reduce cardiovascular risk:
         1. **Weight loss is the most effective lifestyle intervention in hypertensive patients who are overweight or obese**. 5 kg weight loss can lead to up to a 10mm Hg drop in SBP. 10kg weight loss can lead to up to 20mm Hg drop in SBP.
         2. DASH diet reduces BP by 10/5. The Mediterranean diet reduces BP by 7/3.
         3. Limiting ETOH use (2 drinks/day for men and 1 drink/day for women) decreases SBP by 2-4mm Hg.
         4. Regular physical activity (150 minutes/week of moderate intensity exercise or 75 minutes/week of high intensity exercise) decreases SBP by 4-9 mm Hg.
         5. Reducing sodium intake to achieve 24h urine Na excretion of 1500mg/day decreases BP by 7/3.
   2. He is willing to work on changing his diet. How would you counsel him on his diet with the goal of improving his hypertension? What is the evidence behind these recommendations?
      1. Dietary recommendations for hypertension according to 2013 AHA guidelines for lifestyle management to reduce cardiovascular risk: **DASH diet plus sodium restriction (Grade A, LOE A).**
      2. The DASH (Dietary Approaches to Stop Hypertension)-Sodium trial was a 12-week multi-centered trial with 412 individuals with BP 120-159/80-95 mmHg, randomized to a control (typical American) diet and the DASH diet. In both groups, participants were assigned a high sodium (typical US consumption), normal sodium (upper limit of US recommendations), and low sodium diet with a randomized crossover design.^11^
2. Reduction of sodium intake and the DASH diet both significantly lowered blood pressure, with greater effects in combination than alone. Compared to the control diet with high sodium levels, the DASH diet with low sodium led to a mean systolic blood pressure 7 mmHg lower in participants without hypertension, and 11.5mmHg lower in participants with hypertension.
3. **DASH diet is rich in vegetables and fruits and whole grains, and includes moderate intake of low-fat dairy foods, poultry, fish, and nuts, with reduced intake of fats, sugars, and red meat.**
   - - 1. For a 2000 calorie diet: 4-5 daily servings of vegetables and fruit each, 2-3 daily servings of low-fat dairy; 6-8 daily servings of whole grains, 6 or less daily servings of meat/poultry/fish, 4-5 weekly servings of nuts/seeds/legumes, 2-3 daily servings of fats and oils, 0 added sugars.
          1. The DASH diet is similar to previous diets discussed, but also is rich in nutrients that lower blood pressure: potassium, magnesium, calcium, protein, and fiber.
       2. **General tips when counseling about DASH diet**
          1. Add a serving of vegetables to lunch and another at dinner. Can use frozen produce in place of fresh produce.
          2. Replace dessert with a bowl of fruit.
          3. Replace snacks, like chips, with a handful of nuts or seeds.
          4. Make meat a small part of the meal or use beans/legumes instead.
          5. Replace refined grains with whole grain alternatives.
       3. **Lower sodium intake**. The typical American diet includes 3400 mg/day or more of sodium. AHA recommends consuming no more than 2400mg/day. Further reduction to 1500mg/day can yield greater reduction in BP. However, even reducing intake by at least 1000 mg/day will still lower BP. (Grade B, LOE: B).
   1. You provide him with information on the DASH diet and mention that limiting salt will help his blood pressures as well. He says, “But I hardly ever add any salt to my food!” How would you respond?
      1. **Over 75% of the sodium in the US diet comes from food processing, only 10% is added at the table or in cooking^12^**.
      2. The top 3 foods in the US responsible for the most dietary salt intake are bread, processed meats, and pizza.
      3. **General tips to help lower sodium**
         1. Use other spices to flavor foods
         2. Cook at home more often than you eat out
         3. Cut back on pizza, processed meats, refined breads, instant/packaged foods and frozen dinners
         4. Rinse canned beans to remove sodium
4. Ms. Jones is a 59 yo F with BMI 23 and hyperlipidemia. Her last fasting lipid panel shows TC 250, LDL 170, HDL 45, TG 200. She asks you how she can change her diet to improve her health.
   1. How would you respond?
      1. **Dietary recommendations to lower LDL according to the 2013 AHA guidelines for lifestyle management to lower cardiovascular risk: Eat vegetables, fruits, whole grains. Use low fat dairy products, proteins like poultry/fish/legumes/nuts, vegetable oils. Limit sweets, sugar-sweetened beverages, and red meats.** – Grade A, LOE: A.
         1. Note that these are similar suggestions to the heart healthy diet we talked about in question 1. This diet will allow for increased fiber intake, decreased saturated fats and trans fats, and increased intake of polyunsaturated fatty acids (PUFA) and monounsaturated fatty acids (MUFA).
         2. Fiber slows the rate of absorption and digestion, preventing not only a rapid rise in BG but also in lipid levels. It helps keep you full longer.
      2. Meta-analysis of controlled trials of plant-based diets showed that plant-based diets were associated with lower LDL (mean -12.2) compared with omnivorous diets; the association is strongest with vegan diets^13^.
   2. As part of your counseling, you tell her about reducing saturated fats and replacing them with polyunsaturated fatty acids and monounsaturated fatty acids, but she looks confused. She states, “Wait, what? I thought all fats were bad for you.” How would you respond?
      1. **Not all fats are bad for you. Replacing 5% of calories from saturated fats with equivalent calories from MUFA or PUFA is associated with 13 and 27% decreased all-cause mortality**, **respectively,** based on data from Nurses’ Health Study and Health Professionals Follow-up Study.^14^
      2. **Trans fats** - naturally occurring in animal products or artificially produced through an industrial process that adds hydrogen to liquid vegetable oils to create partially hydrogenated oils (used in fried foods, baked goods, processed foods). The FDA no longer allows industries to use partially hydrogenated oils.
      3. **Saturated fats** - decrease the number and activity of LDL receptors, leading to increased serum LDL.
         1. Full-fat dairy, meat, coconut and palm oils, butter, animal lard
         2. Average saturated fat intake in US diet is currently estimated at 11%.
      4. **Polyunsaturated fats** – provide essential fatty acids omega 3 and omega 6 which the body does not produce, as well as antioxidants.
         1. Walnuts, flax, chia
         2. Plant based oils contain a mixture of MUFA and PUFA
         3. Fish with high omega 3 and low mercury levels– salmon, trout, sardines
      5. **Monounsaturated fats**
         1. Plant based oils contain a mixture of MUFA and PUFA
         2. Avocado, nuts

ASSESSMENT

1. A 40 yo F has an LDL of 160 and BMI of 24. Her weight is 140 lbs with waist circumference 33 in. In the last 24h, she ate bacon, pancakes with butter and syrup, hot dog and a salad from a fast food restaurant, and a hamburger with vegetable soup and yogurt. What one lifestyle change would most help to lower LDL?
   1. Use coconut oil instead of butter
   2. Try salmon and trout instead of hamburgers.
      1. This patient is not overweight and has a normal waist circumference so does not necessarily need to focus on weight loss (answer c). Although answer d is appropriate, we should be providing more specific advice on what foods have added sugars and what to replace them with. Answer b would help replace saturated fats with PUFA and MUFA. She eats a lot of processed meats like bacon, hot dogs, and hamburgers, so cutting down on these would be an important goal for her- she could instead try leaner meats and plant-based proteins (legumes, soy, nuts, seeds). Coconut oil contains a high amount of saturated fats (answer a), a better alternative would be olive oil or canola oil.
   3. Lose 10 lbs in the next 6 months.
   4. Eat less added sugars.
2. A 53 yo M with hypertension presents for follow up. His blood pressures range 140-150/70-85. He is frustrated because he is trying to eat healthy in order to avoid needing medication to treat his hypertension. He cooks most of his meals at home; his diet consists mostly of toast and eggs for breakfast, tuna salad with chips for lunch, and lentils and rice for dinner. What one personalized dietary modification could you suggest to this patient?
   1. Don’t add salt at the table to foods.
   2. Eat less processed food.
   3. Replace the side of chips with a side of vegetables for lunch.
      1. This patient is eating minimal amounts of produce. The DASH diet can reduce BP by up to 10/5 and consists of high fruit and vegetable intake; reasonable suggestions would be to work up to this goal by eating a piece of fruit for a snack, adding a side of vegetables to lunch and dinner, etc. While a restricted sodium diet is also indicated for hypertension management, only 10% of dietary sodium intake comes from adding salt while cooking or at the table (answer a); also, there is no indication he is doing that. While cutting back on processed foods is good advice (answer b), he doesn’t seem to be eating a lot of processed foods; also, if you suggest cutting back on foods, you should provide healthier alternatives. A low carb diet (d) is not indicated for hypertension management, although could be an option for short term weight loss depending on patient preferences.
   4. Decrease carbohydrate intake to < 30% of total daily calories.

REFERENCES

1. Bhaskaran K, dos-Santos-Silva I, Leon DA, Douglas IJ, Smeeth L. Association of BMI with overall and cause-specific mortality: a cohort study of 3.6 million adults in the UK. *Lancet Diabetes Endocrinol*. 2018;6(12):944‒953. <https://doi.org/10.1016/s2213-8587(18)30288-2>.
2. National Heart, Lung, and Blood Institute. “According to Waist Circumference.” *Guidelines on Overweight and Obesity: Electronic Textbook.* Retrieved from <https://www.nhlbi.nih.gov/health-pro/guidelines/current/obesity-guidelines/e_textbook/txgd/4142.htm> on June 2020.
3. Micha R, Peñalvo JL, Cudhea F, Imamura F, Rehm CD, Mozaffarian D. Association Between Dietary Factors and Mortality From Heart Disease, Stroke, and Type 2 Diabetes in the United States. JAMA. 2017;317(9):912–924. doi:10.1001/jama.2017.0947
4. Joshipura KJ, Hu FB, Manson JE, Stampfer MJ, Rimm EB, Speizer FE, Colditz G, Ascherio A, Rosner B, Spiegelman D, Willett WC. The effect of fruit and vegetable intake on risk for coronary heart disease. *Ann Intern Med,* 2001, 134(12): 1106-14.
5. Guasch-Ferre M, Liu X, Malik VS, Sun Q, Willett WC, Manson JE, Rexrode KM, Li Y, Hu FB, Bhupathiraju SN. Nut consumption and risk of cardiovascular disease. *Journal of American College of Cardiology,* 2017, 70(20): 2519-32.
6. Mellen PB, Walsh TF, Herrington DM. Whole grain intake and cardiovascular disease: a meta-analysis. *Nutr Metab Cardiovasc Dis,* 2008, 18(4): 283-90.
7. Estruch RE, Ros E, Salas-Salvado J, Covas MI. Primary prevention of cardiovascular disease with a Mediterranean diet. *New England Journal of Medicine,* 2013, 368: 1279-1290.
8. De Lorgeril M, Salen P, Martin JL, Monjaud I, Delaye J, Mamelle N. Mediterranean diet, traditional risk factors, and the rate of cardiovascular complications after myocardial infarction: final report of the Lyon Diet Heart Study. *Circulation,* 1999, 99(6): 779-85.
9. Dinu M, Abbate R, Gensini GF, Casini A, Sofi F. Vegetarian, vegan diets and multiple health outcomes: a systematic review with meta-analysis of observational studies. *Crit Rev Food Sci Nutr,* 2017, 57(17): 3640-3649.
10. Song M, Fung TT, Hu FB, Willett WC, Longo VD, Chan AT, Giovannucci EL. Association of animal and plant protein intake with all-cause and cause-specific mortality. *JAMA Intern Med,* 2016, 176(10): 1453-1463.
11. Sacks FM, Svetkey LP, Vollmer WM, et al. Effects on blood pressure of reduced dietary sodium and the Dietary Approaches to Stop Hypertension (DASH) diet. DASH-Sodium Collaborative Research Group. *N Engl J Med*. 2001;344(1):3‐10. doi:10.1056/NEJM200101043440101
12. Adams KM, Kohlmeier M. “Lifestyle management of hypertension.” *Nutrition in Medicine.* UNC Chapel Hill, May 2010, Reviewed Nov 2014. http://www.nutritioninmedicine.org/portal/
13. Yokoyama Y, Levin SM, Barnard ND. Association between plant-based diets and plasma lipids: a systematic review and meta-analysis. *Nutrition Reviews,* 2017, 75(9): 683-698.
14. Wang DD, Li Y, Chiuve SE, Stampfer MJ, Manson ME, Rimm EB, Willett WC, Hu FB. Association of specific dietary fats with total and cause-specific mortality. *JAMA Intern Med,* 2016, 176(8): 1134-45.
15. Eckel RH et al. 2013 AHA/ACC guideline on lifestyle management to reduce cardiovascular risk: a report of the American College of Cardiology/American Heart Association Task Force on Practice Guidelines. *Journal of the American College of Cardiology,* 63 (25 Part B), 2014: 2960-2984.
16. Jensen MD et al. 2013 AHA/ACC/TOS guideline for the management of overweight and obesity in adults: a report of the American College of Cardiology/American Heart Association Task Force on Practice Guidelines and The Obesity Society. *Journal of the American College of Cardiology,* 63(25 Part B), 2014: 2985-3023.
