# Supplementary material for: A Nutrition Counseling Curriculum to Address Cardiovascular Risk Reduction for Internal Medicine Residents
Source: MedEdPORTAL. 2020 Nov 11;16:11027. doi: 10.15766/mep_2374-8265.11027 (PMC7666832; doi:10.15766/mep_2374-8265.11027)
Supplement: Supplementary file 1 — Session 1 Preceptor Handout.docxSession 1 Resident Handout.docxSession 2 Preceptor Handout.docxSession 2 Resident Handout.docxTake-Home Handout.docxPre-and Postsurvey.docx [file mep_2374-8265.11027-s001.zip › B. Session 1 Resident Handout.docx]

NUTRITION COUNSELING TO REDUCE CARDIOVASCULAR RISK- SESSION 1

OBJECTIVES

1. Improve resident attitudes regarding the role of the primary care provider in nutrition counseling for patients with hypertension, hyperlipidemia, overweight and obesity, and cardiovascular disease.
2. Identify waist circumference as a risk factor for cardiovascular disease.
3. List evidence-based dietary recommendations for the management of overweight and obesity, HTN, HLD, and CVD.
4. Ms. Turner is a 45 yo F with BMI 29. She comes in for routine follow up. Her waist circumference at today’s visit is 37 in. Her blood pressure is 120/70.
   1. What are her risk factors for cardiovascular disease?
   2. How does waist circumference help to risk stratify patients? How do you measure this? How often should you measure waist circumference?
   3. You ask her how she feels about her weight and she replies that she wants to lose weight but isn’t sure where to start. How would you counsel this patient to help her lose weight?
5. You see Ms. Turner for follow up 6 months later. She has since lost 5 lbs and her waist circumference is 35 in. You congratulate her on her success. She thanks you and states that although she has made some dietary changes, she is still worried about her diet. She says, “I mostly drink just water now instead of sweet tea, but is there anything else I should or should not be eating? Heart disease runs in my family and I’m worried about having a heart attack one day.”
   1. Have you provided nutrition counseling to your patients? What barriers have you come across that prevent you from routinely counseling about diet?
   2. What is the benefit of nutrition counseling for cardiovascular risk? What is the role of the PCP in these situations?
   3. What advice would you provide Ms. Turner in response to her question?
6. Mr. Smith is a 52 yo M with HTN and obesity who has a BMI of 36. His BP is 150/90 at today’s visit confirmed on manual recheck. He eats 3 meals/day and favors snack foods like chips and dip. He eats out 2-3 times/week at buffet style restaurants. He has 1 drink with hard liquor before dinner and 2 beers daily. He walks a block to work daily.
   1. What are targets for lifestyle intervention you could work on with this patient? What lifestyle change(s) would provide the greatest reduction in his blood pressure?

- 1. He is willing to work on changing his diet. How would you counsel him on his diet with the goal of improving his hypertension?
  2. You provide him with information on the DASH diet and mention that limiting salt will help his blood pressures as well. He says, “But I hardly ever add any salt to my food!” How would you respond?

1. Ms. Jones is a 59 yo F with BMI 23 and hyperlipidemia. Her last fasting lipid panel shows TC 250, LDL 170, HDL 45, TG 200. She asks you how she can change her diet to improve her health.
   1. How would you respond?
   2. As part of your counseling, you tell her about reducing saturated fats and replacing them with polyunsaturated fatty acids and monounsaturated fatty acids, but she looks confused. She states, “Wait, what? I thought all fats were bad for you.” How would you respond?

ASSESSMENT

1. A 40 yo F has an LDL of 160 and BMI of 24. Her weight is 140 lbs with waist circumference 33 in. In the last 24h, she ate bacon, pancakes with butter and syrup, hot dog and a salad from a fast food restaurant, and a hamburger with vegetable soup and yogurt. What one lifestyle change would most help to lower LDL?
   1. Use coconut oil instead of butter
   2. Try salmon and trout instead of hamburgers.
   3. Lose 10 lbs in the next 6 months.
   4. Eat less added sugars.
2. A 53 yo M with hypertension presents for follow up. His blood pressures range 140-150/70-85. He is frustrated because he is trying to eat healthy in order to avoid needing medication to treat his hypertension. He cooks most of his meals at home; his diet consists mostly of toast and eggs for breakfast, tuna salad with chips for lunch, and lentils and rice for dinner. What one personalized dietary modification could you suggest to this patient?
   1. Don’t add salt at the table to foods.
   2. Eat less processed food.
   3. Replace the side of chips with a side of vegetables for lunch.
   4. Decrease carbohydrate intake to < 30% of total daily calories.

REFERENCES

Adams KM, Kohlmeier M. “Lifestyle management of hypertension.” *Nutrition in Medicine.* UNC Chapel Hill, May 2010, Reviewed Nov 2014. http://www.nutritioninmedicine.org/portal/

Berger S, Raman G, Vishwanathan R, Jacques PF, Johnson EJ. Dietary cholesterol and cardiovascular disease: a systematic review and meta-analysis. *Am J Clin Nutr,* 2015, 102(2): 276-94.

De Lorgeril M, Salen P, Martin JL, Monjaud I, Delaye J, Mamelle N. Mediterranean diet, traditional risk factors, and the rate of cardiovascular complications after myocardial infarction: final report of the Lyon Diet Heart Study. *Circulation,* 1999, 99(6): 779-85.

Dinu M, Abbate R, Gensini GF, Casini A, Sofi F. Vegetarian, vegan diets and multiple health outcomes: a systematic review with meta-analysis of observational studies. *Crit Rev Food Sci Nutr,* 2017, 57(17): 3640-3649.

Eckel RH et al. 2013 AHA/ACC guideline on lifestyle management to reduce cardiovascular risk: a report of the American College of Cardiology/American Heart Association Task Force on Practice Guidelines. *Journal of the American College of Cardiology,* 63 (25 Part B), 2014: 2960-2984.

Estruch RE, Ros E, Salas-Salvado J, Covas MI. Primary prevention of cardiovascular disease with a Mediterranean diet. *New England Journal of Medicine,* 2013, 368: 1279-1290.

Guasch-Ferre M, Liu X, Malik VS, Sun Q, Willett WC, Manson JE, Rexrode KM, Li Y, Hu FB, Bhupathiraju SN. Nut consumption and risk of cardiovascular disease. *Journal of American College of Cardiology,* 2017, 70(20): 2519-32.

Jensen MD et al. 2013 AHA/ACC/TOS guideline for the management of overweight and obesity in adults: a report of the American College of Cardiology/American Heart Association Task Force on Practice Guidelines and The Obesity Society. *Journal of the American College of Cardiology,* 63(25 Part B), 2014: 2985-3023.

Joshipura KJ, Hu FB, Manson JE, Stampfer MJ, Rimm EB, Speizer FE, Colditz G, Ascherio A, Rosner B, Spiegelman D, Willett WC. The effect of fruit and vegetable intake on risk for coronary heart disease. *Ann Intern Med,* 2001, 134(12): 1106-14.

Kohlmeier M, Adams KM, Dong O. “Cholesterol Lowering with Lifestyle Changes.” *Nutrition in Medicine.* UNC Chapel Hill, Dec 2013, Reviewed Nov 2014. <http://www.nutritioninmedicine.org/portal/>

Mellen PB, Walsh TF, Herrington DM. Whole grain intake and cardiovascular disease: a meta-analysis. *Nutr Metab Cardiovasc Dis,* 2008, 18(4): 283-90.

Micha R, Peñalvo JL, Cudhea F, Imamura F, Rehm CD, Mozaffarian D. Association Between Dietary Factors and Mortality From Heart Disease, Stroke, and Type 2 Diabetes in the United States. JAMA. 2017;317(9):912–924. doi:10.1001/jama.2017.0947

Morris S, Adams KM, Kohlmeier M. “Behavior Change Counseling.” *Nutrition in Medicine*. UNC Chapel Hill, Oct 2009, Reviewed Feb 2015. http://www.nutritioninmedicine.org/portal/

Paxton et al. Starting the Conversation: Performance of a brief dietary assessment and intervention tool for health professionals. *Am J Prev Med*, Jan 2011, 40(1), 67-71.

Patterson RE, Sears DD. Metabolic effects of intermittent fasting. *Annual Review of Nutrition,* 2017, 37: 371-393.

Pearlman M, Obert J, Casey L. The association between artificial sweeteners and obesity. *Curr Gastroenterology Rep,* 2017, 19(12): 64.

“Should you try the keto diet?” Harvard Health Letter. *Harvard Health Publishing,* Oct 2018. https://www.health.harvard.edu/staying-healthy/should-you-try-the-keto-diet

Song M, Fung TT, Hu FB, Willett WC, Longo VD, Chan AT, Giovannucci EL. Association of animal and plant protein intake with all-cause and cause-specific mortality. *JAMA Intern Med,* 2016, 176(10): 1453-1463.

U.S. Department of Health and Human Services and U.S. Department of Agriculture. 2015 – 2020 Dietary Guidelines for Americans. 8^th^ Edition. December 2015. Available at <http://health.gov/dietaryguidelines/2015/guidelines/>.

Wang DD, Li Y, Chiuve SE, Stampfer MJ, Manson ME, Rimm EB, Willett WC, Hu FB. Association of specific dietary fats with total and cause-specific mortality. *JAMA Intern Med,* 2016, 176(8): 1134-45.

Yokoyama Y, Levin SM, Barnard ND. Association between plant-based diets and plasma lipids: a systematic review and meta-analysis. *Nutrition Reviews,* 2017, 75(9): 683-698.
