# Supplementary material for: A Nutrition Counseling Curriculum to Address Cardiovascular Risk Reduction for Internal Medicine Residents
Source: MedEdPORTAL. 2020 Nov 11;16:11027. doi: 10.15766/mep_2374-8265.11027 (PMC7666832; doi:10.15766/mep_2374-8265.11027)
Supplement: Supplementary file 1 — Session 1 Preceptor Handout.docxSession 1 Resident Handout.docxSession 2 Preceptor Handout.docxSession 2 Resident Handout.docxTake-Home Handout.docxPre-and Postsurvey.docx [file mep_2374-8265.11027-s001.zip › C. Session 2 Preceptor Handout.docx]

NUTRITION COUNSELING TO REDUCE CARDIOVASCULAR RISK- SESSION 2

OBJECTIVES

For patients with cardiovascular disease, HTN, HLD, overweight and obesity, residents should be able to:

1. Take a focused dietary history
2. Assess stage of change regarding lifestyle behaviors
3. Initiate individualized nutrition counseling in appropriate patients and answer common patient questions about diet
4. Refer appropriate patients to a health care provider specializing in nutrition counseling

*Please leave 5-10 minutes at the end of the pre-clinic conference session to allow residents time to complete the post-curriculum survey.

1. Ms. F is a 33 yo F with BMI 32 and waist circumference 37 in. She wants to start eating healthier. You want to assess her current diet so you can help her target areas for improvement. How would you take a dietary history for this patient?
   1. There is no one validated screening assessment supported by major guidelines to assess dietary risk in primary care settings. The screening tools used for epidemiologic studies (24h food recall, food frequency questionnaire, food diaries) are useful but time consuming; they are meant for the research setting or for use by a health care provider specializing in nutrition.
   2. *Optional:* One example of a brief screening tool is Starting the Conversation^1^: validated in T2DM population and available in the public domain. Higher scores reflect more room for improvement in diet.
   3. You learn that she is eating a lot of refined carbohydrates, like white breads and pasta, and drinking several glasses of soda daily. What advice would you give her?
      1. Replace soda with water or unsweetened beverages. Limit items with high amounts of “added sugars,” which you can look for on the nutrition labels and ingredient list; a simplified recommendation would be to limit items that list a sugar as the first or second ingredient.
         1. Added sugars are sugars and syrups added to foods when processed or prepared. They are not the naturally occurring sugars like those in fruit or milk. Added sugars include corn syrup, sucrose, fructose, glucose, and dextrose. They provide extra calories and no additional nutritional need or benefit.
         2. Observational studies indicate a higher intake of soft drinks are associated with greater caloric intake, higher body weight, and lower intake of essential nutrients.^2^
      2. Replace refined carbohydrates (processed snack foods, baked goods, white breads/pastas/rice) with healthier carbohydrates (fruits, whole grains like brown rice, wild rice, quinoa, oats, rye, barley, bulgur wheat).
         1. Be aware that labels that say “made with whole grain” may still contain refined grains. Choose products that have the whole grain listed first in the ingredient list.
      3. Whole grains contain all parts of the original grain – providing fiber, vitamins, antioxidants, in addition to the carbohydrate. Refined grains contain mostly the inner part of the grain, which is predominantly just the carbohydrate.
         1. Fiber helps slow the breakdown of starch into glucose, allowing for a more steady level of blood sugar levels rather than causing sharp spikes.
         2. As we discussed in Session 1, whole grains as part of a healthy diet can lower cardiovascular risk. In a meta-analysis of seven prospective cohort studies, an average of 2.5 servings of whole grains daily was associated with a 21% lower risk of CVD events^3^.
   4. She then asks, “What about drinking diet soda instead of regular soda?”
      1. It’s likely best to limit use of both added sugars and artificial sweeteners. Data in both animal models and humans suggest that artificial sweeteners appear to alter the microbiome, altering glucose homeostasis. They are associated with increased caloric consumption and weight gain^4^.
   5. She asks next, “What about intermittent fasting? I’ve heard that can be helpful to lose weight.”
      1. Intermittent fasting involves cycles between periods of fasting and unrestricted eating; the thought behind this is that allowing for a fasting state helps improve metabolism and lowers blood sugar levels. A simple form of intermittent fasting would be to eat only during 8 hours of the day (ie 8am-4pm), allowing for a prolonged fasting state overnight. This is also good because nighttime eating is associated with a higher risk of obesity and diabetes.^5^
      2. Intermittent fasting can result in weight loss, although studies have not shown that intermittent fasting is superior to other weight loss methods (ie continuous calorie restriction) in terms of weight loss or compliance rates. Animal studies have shown improvement in metabolic profiles and improved insulin sensitivity, however data in human studies is limited. More long-term high-quality studies are still needed.^6^
      3. Certain populations should not attempt these diets unless closely supervised by a physician and/or dietician: people with insulin-dependent diabetes, history of eating disorders, women who are pregnant or breastfeeding.
   6. She responds, “This sounds like it’s going to be pretty tough…Where am I even supposed to find more information on how to eat healthy?”
      1. *Optional resources:*
         1. Resources on eating healthy when aiming for a healthy weight: <https://www.nhlbi.nih.gov/health/educational/lose_wt/eat/index.htm>
         2. Epic patient instructions (in “references”) on losing weight and/or Mediterranean diet
         3. Recipes: <https://www.cookinglight.com/>
      2. **Refer to a dietician or health coach:**
         1. Any patient that has a disease that can be at least partially managed with dietary modifications and who is ready to learn about making dietary changes can and should be referred to a health care provider who specializes in nutrition. This may or may not be covered by insurance- patients can call their insurance to learn more about how many sessions would be covered, as well as calling the dietician’s office for more information.
         2. How to refer in our EHR at Sites A and B:
            1. “Medical nutrition therapy” – this is a referral to a dietician
            2. A nurse diabetic educator is available at Sites A and B for nutrition counseling in patients with diabetes
            3. “Wellness” referrals for nutrition are available for patients with selected insurance at our institution: this involves follow ups by phone either with a health coach or dietician
      3. Note that referrals should not prevent you as the PCP from initiating counseling and following up on adherence and barriers as needed.
2. For each patient scenario, imagine you are providing nutrition counseling to your patient. Assess the patient’s stage of change and give an example of an appropriate response. As you go, fill out the table below.

Transtheoretical Model, Stages of Change

The Transtheoretical Model or Stages of Change Model was developed by Prochaska and DiClemente in the 1970s based on studies examining why some people were capable of quitting smoking. The model assumes that people change habits and behaviors not quickly or decisively but through a cyclical process.^7^ In other words, people are not going to change their behaviors unless they are ready to do so. This model is useful when assessing behaviors that affect health to help engage in a more productive conversation with your patient, especially when combined with motivational interviewing techniques.^8^

| Stage of Change | Description | Provider response |
| --- | --- | --- |
| Precontemplation | Patient unaware that behavior has negative consequences, focuses on the cons of changing the behavior | Reflect back on the patient’s statements expressing their reasons for continuing the same behavior; this is called sustain talk. Encourage more open-ended exploration. (It sounds like its more effort than you are willing to make right now.) |
| Contemplation | Patient aware of problem, thinking about change but not committed; thoughtful consideration of the pros and cons of changing behavior, but still feels ambivalent towards changing behavior | Reflect back the patient’s sustain talk. Help patient resolve ambivalence toward change (It sounds like this is becoming a stronger priority for you…What would need to change for you to start thinking about this more seriously?) |
| Preparation | Aware of benefits of change and intending to change, taking small steps toward change | Provide education on specifics of behavior modification and help set goals (Let’s see how you can reduce the amount of sugary foods you eat in a day.) |
| Action | Patient has changed behavior in last 6 months and intends to continue | Provide support and guidance; address barriers (It’s great that you’re working so hard. What problems have you faced so far? How have you solved them?) |
| Maintenance | Patient has changed behavior for 6 months or more | Assess with relapse control (What strategies will be helpful the next time you feel tempted?) |

1. A 65 yo M with hyperlipidemia comes in for follow up. His LDL is 170 and total cholesterol is 240. You want to help him with lifestyle management of his conditions. You tell him “I’m concerned your high cholesterol puts you at risk for heart disease.” He says, “I’m so worried about that. My dad died of a heart attack when he was 65, which is how old I am right now. I have been thinking about eating healthier…”
2. Contemplation.
3. “That’s great to hear. I definitely think making some dietary changes could help you lower your cholesterol and reduce your risk for heart disease. What would need to happen for you to improve your diet?”
4. A 47 yo F with HTN who you are following up with after recommending DASH diet at last visit 1 month ago. BP 145/94. She says “The diet has been going pretty well. I’ve cut back to one glass of wine per day and I’ve replaced my dairy with low-fat alternatives; I’ve also switched my frozen dinners to low-sodium options. I’m having trouble getting enough fruits and vegetables though. They are just so expensive!”
5. Action
6. “That’s so great that you’ve already made those big changes! Perhaps try frozen fruits and vegetables if you are having trouble affording fresh produce.” Frozen fruits and vegetables are still healthy options if fresh produce is not available, in season, or affordable. They retain the overall nutritional content of their fresh counterparts.
7. “Although switching to low sodium frozen dinners is a first step, try to cook more of your dinners at home. Maybe meal prep for your dinners a few times per week if you don’t have time to cook every night.”
8. Any healthy eating pattern, including the DASH diet, should limit intake of processed foods including packaged, pre-prepared meals.
9. A 55 yo F with BMI 30, hyperlipidemia, hypertension and prediabetes comes in for follow up. Your goal is to discuss weight loss.
10. How do you bring up the topic of weight with your patients?
    - - 1. This can certainly be a sensitive subject for some patients, however most patients do want their providers to discuss weight management with them. Ultimately the way you bring up this topic depends on your relationship with your patient. One safe opening phrase would be, “How do you feel about your weight?” In addition, tying their weight to the health risks it imposes helps normalize the discussion, similar to the way you talk about hypertension or diabetes to your patients.
11. You ask the patient how they feel about trying to lose weight. She says, “What’s the point? I’m fine with where I’m at right now.”
12. Precontemplation.
13. “It sounds like you see no reasons to change. Can you explain that more for me?”
14. At the patient’s next follow up appointment, she tells you her sister recently had a heart attack. She says, “That really woke me up and made me more worried about my own health. You told me last time that losing weight would help me be healthier—I’ve been thinking about trying the keto diet to lose weight. What do you think?”
15. She has moved to the preparation stage.
16. “I’m so sorry to hear about your sister…I would love to help you make a plan to lose weight. The keto diet is popular but often leads to a high intake of saturated fats and is likely not sustainable in the long term… Why don’t we take a look at your diet and see where we can make a few modifications?”
17. A ketogenic diet severely restricts carbohydrates and relies on fat for the majority of daily calories. It was intended for the treatment of epilepsy but has gained popularity for its purported weight loss results. While it may lead to weight loss in the short term, it is likely not sustainable, and the long-term effects have not been studied. In addition, it leads to increases in saturated fats.^9^
18. A 55 yo M with CAD comes to your office for routine follow up. He has been working on incorporating a Mediterranean diet pattern into his diet for the past 8 months. You congratulate him on the changes he’s made. He replies, “Thanks, I’m feeling great! I still crave my bagel sandwich with bacon, egg, and cheese in the mornings, though.”
19. Maintenance
20. “It can be so hard to cut down on the food we have grown up to love eating and your favorite sandwich is still okay to eat occasionally. However, all processed meats, including bacon, are not only unhealthy, they are also considered carcinogenic by the WHO. And bagels, while they don’t taste sweet, still raise your blood sugar and increase your risk for diabetes. If you want to make a sandwich to eat more regularly, how about using whole grain bread instead of a bagel and avocado or tomato instead of bacon?”
21. A 60 yo M with obesity comes in for routine follow up. He has lost 15 lbs in the past year. You congratulate him on his weight loss and he says “It hasn’t been easy! I still have trouble eating healthy when I eat out at restaurants.”
22. Maintenance
23. That’s definitely challenging. What if you limit the amount of times you eat out so you don’t have to feel as guilty when you do eat at a restaurant? When you do eat out, try these tips:
24. Ask for food to be prepared with less salt and sauces/dressings to be served on the side.
25. Ask the server to not bring the bread basket/chips at the beginning of the meal. Or, take a portion and move the basket away from you so you are not as tempted.
26. If you want dessert, ask for a bowl of fruit.

ASSESSMENT

1. Which of these statements shows a patient in the contemplation stage of change?
   1. I know I need to lose weight but now isn’t a good time – I have too many other things going on.
      1. Contemplation – Patient is aware of her weight as a health issue, however is not yet committed to change.
   2. I’ve been cutting back on soda for a month now as an attempt to start eating healthier.
      1. Action – Patient is actively taking steps toward change.
   3. My wife tells me I should eat better for my high blood pressure, but I’ve been eating this way for years and I feel fine.
      1. Precontemplation- Patient is unaware of his poor diet and its implications toward his hypertension.
   4. I want to change my diet to help lower my blood pressure.
      1. Preparation – Patient is aware of the benefits of changing her diet and is intending to do so.
2. You are following up with a 40 yo M patient with BMI 33, hypertension, and hyperlipidemia. You tell the patient you are concerned her weight puts her at risk for heart disease, she says, “I can’t lose weight and I’m tired of trying.” What is the most appropriate next step?
   1. Give the patient handouts on how to lose 1-2 pounds per week.
   2. Point out her negative attitude and tell her to lose weight.
   3. Refer the patient to a dietician for weight loss counseling.
   4. Reflect on her frustration with prior attempts to lose weight.
      1. This patient is expressing frustration about apparent past attempts to lose weight, so referring to a dietician (answer c) or providing a handout on weight loss (answer a) prior to reflecting on that frustration would likely not be helpful. Pointing out her negative attitude and simply telling her to lose weight (answer b) could increase her frustration. We should address barriers she has faced before with weight loss first.
3. You are seeing a 45 yo F patient with recently diagnosed hypertension. You assess she is in the contemplation stage of change regarding dietary changes to control her blood pressure. What is the most appropriate next step?
   1. Give her a handout on the DASH diet.
   2. Refer the patient to a dietician.
   3. Address ambivalence towards dietary modifications.
      1. If the patient is contemplating dietary changes toward hypertension, we should first help to resolve any ambivalence toward change and ask her about any barriers she is facing preventing her from changing her diet. Answers a, b, and c would all be appropriate steps once we have addressed her ambivalence towards change.
   4. Tell her to follow a 2g sodium diet.

*Please provide the residents with the take-home handout after you have completed this session. *Instruct residents to complete the post-curriculum survey.

REFERENCES

1. Paxton et al. Starting the Conversation: Performance of a brief dietary assessment and intervention tool for health professionals. *Am J Prev Med*, Jan 2011, 40(1), 67-71. doi: 10.1016/j.amepre.2010.10.009.
2. Johnson RK, Appel LJ, Brands M, et al. Dietary sugars intake and cardiovascular health: a scientific statement from the American Heart Association. *Circulation*. 2009;120(11):1011‐1020. doi:10.1161/CIRCULATIONAHA.109.192627
3. Mellen PB, Walsh TF, Herrington DM. Whole grain intake and cardiovascular disease: a meta-analysis. *Nutr Metab Cardiovasc Dis,* 2008, 18(4): 283-90.
4. Pearlman M, Obert J, Casey L. The association between artificial sweeteners and obesity. *Curr Gastroenterology Rep,* 2017, 19(12): 64.
5. Tello M. “Intermittent fasting: surprising update.” Harvard Health Blog*. Harvard Health Publishing,* Feb 2020. <https://www.health.harvard.edu/blog/intermittent-fasting-surprising-update-2018062914156>
6. Patterson RE, Sears DD. Metabolic effects of intermittent fasting. *Annual Review of Nutrition,* 2017, 37: 371-393.
7. Morris S, Adams KM, Kohlmeier M. “Behavior Change Counseling.” *Nutrition in Medicine*. UNC Chapel Hill, Oct 2009, Reviewed Feb 2015. <http://www.nutritioninmedicine.org/portal/>
8. Center for Substance Abuse Treatment. Enhancing Motivation for Change in Substance Abuse Treatment. *Treatment Improvement Protocol (TIP) Series, No. 35*, *Chapter 3: Motivational Interviewing as a Counseling Style*. Rockville (MD): Substance Abuse and Mental Health Services Administration, US. 1999. https://www.ncbi.nlm.nih.gov/books/NBK64964/
9. “Should you try the keto diet?” Harvard Health Letter. *Harvard Health Publishing,* Oct 2018. https://www.health.harvard.edu/staying-healthy/should-you-try-the-keto-diet
