# Supplementary material for: A Nutrition Counseling Curriculum to Address Cardiovascular Risk Reduction for Internal Medicine Residents
Source: MedEdPORTAL. 2020 Nov 11;16:11027. doi: 10.15766/mep_2374-8265.11027 (PMC7666832; doi:10.15766/mep_2374-8265.11027)
Supplement: Supplementary file 1 — Session 1 Preceptor Handout.docxSession 1 Resident Handout.docxSession 2 Preceptor Handout.docxSession 2 Resident Handout.docxTake-Home Handout.docxPre-and Postsurvey.docx [file mep_2374-8265.11027-s001.zip › D. Session 2 Resident Handout.docx]

NUTRITION COUNSELING TO REDUCE CARDIOVASCULAR RISK- SESSION 2

OBJECTIVES

For patients with cardiovascular disease, HTN, HLD, overweight and obesity, residents should be able to:

1. Take a focused dietary history
2. Assess stage of change regarding lifestyle behaviors
3. Initiate individualized nutrition counseling in appropriate patients and answer common patient questions about diet
4. Refer appropriate patients to a health care provider specializing in nutrition counseling
5. Ms. F is a 33 yo F with BMI 32 and waist circumference 37 in. She wants to start eating healthier. You want to assess her current diet so you can help her target areas for improvement.
   1. How would you take a dietary history for this patient?
   2. You learn that she is eating a lot of refined carbohydrates, like white breads and pasta, and drinking several glasses of soda daily. What advice would you give her?
   3. She then asks, “What about drinking diet soda instead of regular soda?”
   4. She asks next, “What about intermittent fasting? I’ve heard that can be helpful to lose weight.”
   5. She responds, “This sounds like it’s going to be pretty tough…Where am I even supposed to find more information on how to eat healthy?”
6. For each patient scenario, imagine you are providing nutrition counseling to your patient. Assess the patient’s stage of change and give an example of an appropriate response. As you go, fill out the table below.

Transtheoretical Model, Stages of Change. Adapted from Morris et al, 2009.

| Stage of Change | Description | Provider response |
| --- | --- | --- |
|  |  |  |
|  |  |  |
|  |  |  |
|  |  |  |
|  |  |  |

1. A 65 yo M with hyperlipidemia comes in for follow up. His LDL is 170 and total cholesterol is 240. You want to help him with lifestyle management of his conditions. You tell him “I’m concerned your high cholesterol puts you at risk for heart disease.” He says, “I’m so worried about that. My dad died of a heart attack when he was 65, which is how old I am right now. I have been thinking about eating healthier…”
2. A 47 yo F with HTN who you are following up with after recommending DASH diet at last visit 1 month ago. BP 145/94. She says “The diet has been going pretty well. I’ve cut back to one glass of wine per day and I’ve replaced my dairy with low-fat alternatives; I’ve also switched my frozen dinners to low-sodium options. I’m having trouble getting enough fruits and vegetables though. They are just so expensive!”
3. A 55 yo F with BMI 30, hyperlipidemia, hypertension and prediabetes comes in for follow up. Your goal is to discuss weight loss.
4. How do you bring up the topic of weight with your patients?
5. You ask the patient how they feel about trying to lose weight. She says, “What’s the point? I’m fine with where I’m at right now.”
6. At the patient’s next follow up appointment, she tells you her sister recently had a heart attack. She says, “That really woke me up and made me more worried about my own health. You told me last time that losing weight would help me be healthier—I’ve been thinking about trying the keto diet to lose weight. What do you think?”
7. A 55 yo M with CAD comes to your office for routine follow up. He has been working on incorporating a Mediterranean diet pattern into his diet for the past 8 months. You congratulate him on the changes he’s made. He replies, “Thanks, I’m feeling great! I still crave my bagel sandwich with bacon, egg, and cheese in the mornings, though.”
8. A 60 yo M with obesity comes in for routine follow up. He has lost 15 lbs in the past year. You congratulate him on his weight loss and he says “It hasn’t been easy! I still have trouble eating healthy when I eat out at restaurants.”

ASSESSMENT

1. Which of these statements shows a patient in the contemplation stage of change?
   1. I know I need to lose weight but now isn’t a good time – I have too many other things going on.
   2. I’ve been cutting back on soda for a month now as an attempt to start eating healthier.
   3. My wife tells me I should eat better for my high blood pressure, but I’ve been eating this way for years and I feel fine.
   4. I want to change my diet to help lower my blood pressure.
2. You are following up with a 40 yo M patient with BMI 33, hypertension, and hyperlipidemia. You tell the patient you are concerned her weight puts her at risk for heart disease, she says, “I can’t lose weight and I’m tired of trying.” What is the most appropriate next step?
   1. Give the patient handouts on how to lose 1-2 pounds per week.
   2. Point out her negative attitude and tell her to lose weight.
   3. Refer the patient to a dietician for weight loss counseling.
   4. Reflect on her frustration with prior attempts to lose weight.
3. You are seeing a 45 yo F patient with recently diagnosed hypertension. You assess she is in the contemplation stage of change regarding dietary changes to control her blood pressure. What is the most appropriate next step?
   1. Give her a handout on the DASH diet.
   2. Refer the patient to a dietician.
   3. Address ambivalence towards dietary modifications.
   4. Tell her to follow a 2g sodium diet.

REFERENCES

Adams KM, Kohlmeier M. “Lifestyle management of hypertension.” *Nutrition in Medicine.* UNC Chapel Hill, May 2010, Reviewed Nov 2014. http://www.nutritioninmedicine.org/portal/

Berger S, Raman G, Vishwanathan R, Jacques PF, Johnson EJ. Dietary cholesterol and cardiovascular disease: a systematic review and meta-analysis. *Am J Clin Nutr,* 2015, 102(2): 276-94.

De Lorgeril M, Salen P, Martin JL, Monjaud I, Delaye J, Mamelle N. Mediterranean diet, traditional risk factors, and the rate of cardiovascular complications after myocardial infarction: final report of the Lyon Diet Heart Study. *Circulation,* 1999, 99(6): 779-85.

Dinu M, Abbate R, Gensini GF, Casini A, Sofi F. Vegetarian, vegan diets and multiple health outcomes: a systematic review with meta-analysis of observational studies. *Crit Rev Food Sci Nutr,* 2017, 57(17): 3640-3649.

Eckel RH et al. 2013 AHA/ACC guideline on lifestyle management to reduce cardiovascular risk: a report of the American College of Cardiology/American Heart Association Task Force on Practice Guidelines. *Journal of the American College of Cardiology,* 63 (25 Part B), 2014: 2960-2984.

Estruch RE, Ros E, Salas-Salvado J, Covas MI. Primary prevention of cardiovascular disease with a Mediterranean diet. *New England Journal of Medicine,* 2013, 368: 1279-1290.

Guasch-Ferre M, Liu X, Malik VS, Sun Q, Willett WC, Manson JE, Rexrode KM, Li Y, Hu FB, Bhupathiraju SN. Nut consumption and risk of cardiovascular disease. *Journal of American College of Cardiology,* 2017, 70(20): 2519-32.

Jensen MD et al. 2013 AHA/ACC/TOS guideline for the management of overweight and obesity in adults: a report of the American College of Cardiology/American Heart Association Task Force on Practice Guidelines and The Obesity Society. *Journal of the American College of Cardiology,* 63(25 Part B), 2014: 2985-3023.

Joshipura KJ, Hu FB, Manson JE, Stampfer MJ, Rimm EB, Speizer FE, Colditz G, Ascherio A, Rosner B, Spiegelman D, Willett WC. The effect of fruit and vegetable intake on risk for coronary heart disease. *Ann Intern Med,* 2001, 134(12): 1106-14.

Kohlmeier M, Adams KM, Dong O. “Cholesterol Lowering with Lifestyle Changes.” *Nutrition in Medicine.* UNC Chapel Hill, Dec 2013, Reviewed Nov 2014. <http://www.nutritioninmedicine.org/portal/>

Mellen PB, Walsh TF, Herrington DM. Whole grain intake and cardiovascular disease: a meta-analysis. *Nutr Metab Cardiovasc Dis,* 2008, 18(4): 283-90.

Micha R, Peñalvo JL, Cudhea F, Imamura F, Rehm CD, Mozaffarian D. Association Between Dietary Factors and Mortality From Heart Disease, Stroke, and Type 2 Diabetes in the United States. JAMA. 2017;317(9):912–924. doi:10.1001/jama.2017.0947

Morris S, Adams KM, Kohlmeier M. “Behavior Change Counseling.” *Nutrition in Medicine*. UNC Chapel Hill, Oct 2009, Reviewed Feb 2015. http://www.nutritioninmedicine.org/portal/

Paxton et al. Starting the Conversation: Performance of a brief dietary assessment and intervention tool for health professionals. *Am J Prev Med*, Jan 2011, 40(1), 67-71.

Patterson RE, Sears DD. Metabolic effects of intermittent fasting. *Annual Review of Nutrition,* 2017, 37: 371-393.

Pearlman M, Obert J, Casey L. The association between artificial sweeteners and obesity. *Curr Gastroenterology Rep,* 2017, 19(12): 64.

“Should you try the keto diet?” Harvard Health Letter. *Harvard Health Publishing,* Oct 2018. https://www.health.harvard.edu/staying-healthy/should-you-try-the-keto-diet

Song M, Fung TT, Hu FB, Willett WC, Longo VD, Chan AT, Giovannucci EL. Association of animal and plant protein intake with all-cause and cause-specific mortality. *JAMA Intern Med,* 2016, 176(10): 1453-1463.

U.S. Department of Health and Human Services and U.S. Department of Agriculture. 2015 – 2020 Dietary Guidelines for Americans. 8^th^ Edition. December 2015. Available at <http://health.gov/dietaryguidelines/2015/guidelines/>.

Wang DD, Li Y, Chiuve SE, Stampfer MJ, Manson ME, Rimm EB, Willett WC, Hu FB. Association of specific dietary fats with total and cause-specific mortality. *JAMA Intern Med,* 2016, 176(8): 1134-45.

Yokoyama Y, Levin SM, Barnard ND. Association between plant-based diets and plasma lipids: a systematic review and meta-analysis. *Nutrition Reviews,* 2017, 75(9): 683-698.
