# Supplementary material for: A Nutrition Counseling Curriculum to Address Cardiovascular Risk Reduction for Internal Medicine Residents
Source: MedEdPORTAL. 2020 Nov 11;16:11027. doi: 10.15766/mep_2374-8265.11027 (PMC7666832; doi:10.15766/mep_2374-8265.11027)
Supplement: Supplementary file 1 — Session 1 Preceptor Handout.docxSession 1 Resident Handout.docxSession 2 Preceptor Handout.docxSession 2 Resident Handout.docxTake-Home Handout.docxPre-and Postsurvey.docx [file mep_2374-8265.11027-s001.zip › E. Take-Home Handout.docx]

NUTRITION TO REDUCE CARDIOVASCULAR RISK

1. Follow a healthy eating pattern at an appropriate calorie level
   1. Choose fruits, vegetables, legumes, nuts/seeds and vegetable/olive oils. Frozen fruits/veggies count too!
   2. Limit consumption of processed food, sugar-sweetened beverages, processed meats and red meats
   3. Provide healthier alternatives when cutting down or cutting out food groups. Consider cultural, financial and personal preferences.
2. 2013 AHA guidelines to reduce overweight and obesity: Achieve a negative calorie balance via exercise and reduced caloric intake
   1. No one plan (low carbohydrate vs low fat) is best – individualize to disease states, patient preferences, and cultural preferences
   2. Aim for at least a 500-750 kcal/day energy deficit
   3. Physical activity- 150-300 min/week moderate intensity exercise or 75-150 min/week of vigorous intensity with at least 2 sessions/week of resistance/muscle-strengthening activity
      1. Exercise is the best predictor of long-term weight loss and weight maintenance, can also redistribute abdominal adiposity
3. Tips for counseling your patients about nutrition
   1. Assess their motivation to change their diet
   2. Establish nutrition as a priority for good health
   3. Identify one action item per visit and document it so you can revisit it later
   4. Consider referral to: Nutritionists, Dieticians, Health coaches
   5. When suggesting to patients to cut down or cut out a food group, provide healthier alternatives
   6. Generally, supplements cannot replace the benefits of whole foods. Supplements can be helpful when there is a specific vitamin deficiency (Vitamin D, B12, etc.)

| Type of diet | Description | Evidence | Optional resources |
| --- | --- | --- | --- |
| Mediterranean dietary pattern | - Whole grains - Fruits and vegetables (a variety from all subgroups/colors) - Proteins (seafood, legumes, nuts/seeds) - Olive oil as the predominant oil for cooking - Moderate intake of poultry, eggs, dairy - Limit red meat, saturated fats, trans fats, added sugars, and sodium | - Each daily serving increase in fruits or vegetables: 4% decreased risk for coronary heart disease (green leafy vegetables: 23% decreased risk) - Avg 2.5 servings of whole grains daily: 21% decreased risk of CVD events - 5 or more weekly servings of nuts: 14% decreased risk for cardiovascular disease - PREDIMED primary prevention trial: RCT of > 7000 patients showed decreased rates of MI, CVA, CV death by 28-30% at 5 years | https://health.gov/dietaryguidelines/2015/guidelines/appendix-4/  Epic clinical references for “Mediterranean diet” |
| Plant-based dietary pattern | - Similar to (1), except for no intake of meat or seafood - Limited or no intake of dairy and eggs | - Substitution of animal protein for plant protein: lower all-cause mortality, particularly when substituting for processed red meat (HR 0.66) - Meta-analysis of vegetarian and vegan diets: 25% reduced risk for ischemic heart disease | https://health.gov/dietaryguidelines/2015/guidelines/appendix-5/  https://www.forksoverknives.com/ |
| DASH diet | For a 2000-calorie diet:   - 4-5 servings of vegetables and fruit EACH - 2-3 servings of low-fat dairy - 6-8 servings of whole grains - 6 or less servings of meat/fish - 2-3 servings of fats/oils - 4-5 weekly servings of legumes/nuts/seeds - 0 added sugars | - Reduces blood pressure by up to 10/5 in patients with hypertension, independent of sodium intake or weight loss | https://www.nhlbi.nih.gov/health-topics/dash-eating-plan |
