# Supplementary material for: A Nutrition Counseling Curriculum to Address Cardiovascular Risk Reduction for Internal Medicine Residents
Source: MedEdPORTAL. 2020 Nov 11;16:11027. doi: 10.15766/mep_2374-8265.11027 (PMC7666832; doi:10.15766/mep_2374-8265.11027)
Supplement: Supplementary file 1 — Session 1 Preceptor Handout.docxSession 1 Resident Handout.docxSession 2 Preceptor Handout.docxSession 2 Resident Handout.docxTake-Home Handout.docxPre-and Postsurvey.docx [file mep_2374-8265.11027-s001.zip › F. Pre-and Postsurvey.docx]

SURVEY

Please provide a unique, anonymous identifier: State the first initial of your last name followed by the last 4 digits of your cell phone number (Ex. John Smith, 412-624-1570, would be S1570): ____________

PART ONE: DEMOGRAPHICS

1. Select your gender
   1. Male
   2. Female
   3. Other
2. What is your age? ____________
3. Select your PGY level
   1. PGY1
   2. PGY2
   3. PGY3
4. What training have you had regarding nutrition prior to residency? (Select All)
   1. A dedicated nutrition course in medical school
   2. An undergraduate degree in nutrition science, nutrition dietetics, etc.
   3. A graduate degree in nutrition science, nutrition dietetics, etc.
   4. Other – please describe: ___________________________________
5. What is your intended career choice at this time?
   1. General Medicine/Geriatrics/Palliative Care
   2. Cardiology
   3. Gastroenterology
   4. Hematology/Oncology
   5. Rheumatology
   6. Endocrinology
   7. Pulmonology/Critical Care
   8. Infectious Disease
   9. Nephrology
   10. Undecided

PART TWO: BELIEFS AND BEHAVIORS REGARDING NUTRITION COUNSELING

1. Please indicate what you believe to be the most appropriate statement as it pertains to your continuity clinic patients with each of the following cardiovascular risk factors:

Obesity

1. It is the responsibility of the primary care provider to identify and refer appropriate patients for nutrition counseling.
2. It is the responsibility of the primary care provider to provide nutrition counseling.
3. The role of the primary care provider regarding nutrition counseling depends on the severity of cardiovascular risk for each patient.

Hypertension

1. It is the responsibility of the primary care provider to identify and refer appropriate patients for nutrition counseling.
2. It is the responsibility of the primary care provider to provide nutrition counseling.
3. The role of the primary care provider regarding nutrition counseling depends on the severity of cardiovascular risk for each patient.

Hyperlipidemia

1. It is the responsibility of the primary care provider to identify and refer appropriate patients for nutrition counseling.
2. It is the responsibility of the primary care provider to provide nutrition counseling.
3. The role of the primary care provider regarding nutrition counseling depends on the severity of cardiovascular risk for each patient.

Diabetes

1. It is the responsibility of the primary care provider to identify and refer appropriate patients for nutrition counseling.
2. It is the responsibility of the primary care provider to provide nutrition counseling.
3. The role of the primary care provider regarding nutrition counseling depends on the severity of cardiovascular risk for each patient.
4. Please indicate the frequency to which you perform the following items as it pertains to your continuity clinic patients with each of the following cardiovascular risk factors:

OBESITY

|  | Always | Most of the time | Sometimes | Rarely | Never |
| --- | --- | --- | --- | --- | --- |
| For patients with elevated BMI, I list their weight as a separate problem and/or diagnosis in their chart. |  |  |  |  |  |
| For patients with BMI 25-35, I measure waist circumference for additional risk stratification. |  |  |  |  |  |
| I take a dietary history for my patients. |  |  |  |  |  |
| I use a validated dietary assessment tool when taking a dietary history |  |  |  |  |  |
| I provide nutrition counseling to appropriate patients in my primary care clinic. |  |  |  |  |  |
| I refer appropriate patients in my primary care clinic for nutrition counseling. |  |  |  |  |  |

HYPERTENSION

|  | Always | Most of the time | Sometimes | Rarely | Never |
| --- | --- | --- | --- | --- | --- |
| I take a dietary history for my patients. |  |  |  |  |  |
| I use a validated dietary assessment tool when taking a dietary history |  |  |  |  |  |
| I provide nutrition counseling to appropriate patients in my primary care clinic. |  |  |  |  |  |
| I refer appropriate patients in my primary care clinic for nutrition counseling. |  |  |  |  |  |

HYPERLIPIDEMIA

|  | Always | Most of the time | Sometimes | Rarely | Never |
| --- | --- | --- | --- | --- | --- |
| I take a dietary history for my patients. |  |  |  |  |  |
| I use a validated dietary assessment tool when taking a dietary history |  |  |  |  |  |
| I provide nutrition counseling to appropriate patients in my primary care clinic. |  |  |  |  |  |
| I refer appropriate patients in my primary care clinic for nutrition counseling. |  |  |  |  |  |

PART THREE: MEDICAL KNOWLEDGE

The following questions were adapted from online modules.^1,2^

1. Which one of the following individuals has a higher risk for morbidity and mortality related to their weight?
   1. Male with BMI = 24, waist circumference = 41 in
   2. Female with BMI = 26, waist circumference = 33 in
   3. Male with BMI = 28, waist circumference = 41 in
   4. Female with BMI = 28, waist circumference = 33 in
2. A 47-year-old male with obesity is seeking advice on weight loss diets. Which of the following is the most appropriate recommendation?
   1. He should reduce his caloric intake from fat to 10% of total calories or less.
   2. He should avoid any commercial weight loss programs as they have not been studied.
   3. Either a low carb or low fat diet may be appropriate as the rates of weight loss are the same at 6 and 12 months.
   4. He should follow a very low calorie diet that is limited to 800 calories/day as this strategy would enable him to achieve the greatest sustained weight loss in the long term.
3. A 53-year-old obese male weighing 107kg without other comorbidities is looking for a simple effective dietary intervention to lose weight. Which of the following is a true statement?
   1. Losing 6 kg over 6 months is an unrealistic goal
   2. Losing at most 3 kg over 3 months could be expected with a gradual regain
   3. Losing 8kg over 6 months is a reasonable goal
   4. Losing 3 kg over 6 months is the best case scenario
4. A 40-year-old female patient with obesity states she is ready to try losing weight. You recommend she start a plant-based diet and provide links with new recipes to try. What step did you leave out of this counseling session?
   1. Collaborate with the patient to choose a weight loss plan
   2. Refer patient to dietician before providing advice
   3. Determine whether she understands the health risks of obesity
   4. Address barriers to preparing to change her diet
5. A 42-year-old male with hypertension, on HCTZ, and BMI of 28 presents for follow up. Upon assessing lifestyle behaviors, the patient reveals he frequently eats canned soups, processed meat, frozen dinners, and packaged foods. He is willing to work with you on changing his eating habits. Which of the following is the most appropriate advice to give him?
   1. Don’t add salt to your food at the table or when you cook.
   2. Reduce your portion sizes at meals and cut down on snacks.
   3. Start cooking more at home; replace processed meat with fresh, lean meat.
   4. Cut down on your salt intake.
6. A 35-year-old female with hypertension, on HCTZ, obesity and chronic headaches presents as a new patient to establish care. Her BP is 140/90. She works two retail jobs 7 days/week and eats lunch from a fast food restaurant, usually a burger. She is willing to work with you on changing her eating patterns. Which of the following is the most appropriate advice to give her?
   1. Limit alcohol consumption to 1 drink/day
   2. Reduce daily sodium intake to < 1500mg/day
   3. Increase consumption of omega 3 fatty acids and decrease saturated fats
   4. Pack a lunch with fruit, vegetables, and low-fat dairy
   5. Add 5mg lisinopril daily and check a BMP in 1 week

References

1. Adams KM, Kohlmeier M, Briggs Early K. Effective weight loss strategies for adults. *Nutrition in Medicine.* 2013. <http://www.nutritioninmedicine.org/portal/>
2. Gudzune K, Chaudry ZW, Clark JM. Module: Obesity and overweight: diagnosis and management. *Physician Education and Assessment Center: Internal Medicine Curriculum.* 2018*.* <https://ilc.peaconline.org/>
