# Supplementary material for: A Nutrition Counseling Curriculum to Address Cardiovascular Risk Reduction for Internal Medicine Residents
Source: MedEdPORTAL. 2020 Nov 11;16:11027. doi: 10.15766/mep_2374-8265.11027 (PMC7666832; doi:10.15766/mep_2374-8265.11027)
Supplement: Supplementary file 1 — Session 1 Preceptor Handout.docxSession 1 Resident Handout.docxSession 2 Preceptor Handout.docxSession 2 Resident Handout.docxTake-Home Handout.docxPre-and Postsurvey.docx [file mep_2374-8265.11027-s001.zip › _Educational_Summary_Report_11027.pdf]

MedEdPORTAL® The AAMC Journal of Teaching and Learning Resources

Original Publication Open Access

A Nutrition Counseling Curriculum to Address Cardiovascular Risk Reduction for Internal Medicine Residents

Seema Jain, MD*, Robert Feldman, MS, Andrew D. Althouse, PhD, Carla Spagnoletti, MD, Siobhan Proksell, MD

*Corresponding author: seema.jain@utsouthwestern.edu

Abstract

Introduction: Primary care providers play a critical role in reducing patients' risk for cardiovascular disease, including providing dietary counseling. However, few physicians feel adequately trained to provide this counseling, and most internal medicine (IM) residencies do not offer nutrition education. **Methods:** We created an interactive, case-based activity for IM residents to improve the delivery of nutrition counseling to patients with hypertension, hyperlipidemia, overweight, and obesity. The curriculum was given over two in-person small-group sessions facilitated by physician preceptors. It reviewed evidence for relevant dietary patterns, provided resources for dietary referrals, and allowed residents to practice counseling based on a patient's stage of behavioral change. **Results:** Residents completed electronic surveys prior to curriculum implementation, immediately after, and 2 months after completion of the curriculum. Aggregate percent correct scores of knowledge questions improved significantly in the immediate postsurvey ($n = 24$ paired responses, $p = .004$). We also reviewed electronic health records of patients with body mass index ≥ 25, hypertension, or hyperlipidemia who were seen in our resident clinics 2 months prior ($n = 503$) and 2 months after ($n = 473$) curriculum delivery. Residents' documented nutrition counseling increased from 35% to 41% (odds ratio, 1.27; 95% CI, 0.97-1.67; $p = .085$). **Discussion:** We demonstrated improved knowledge of nutrition interventions to reduce cardiovascular risk and reported improvement of resident-provided nutrition counseling for appropriate patients. This activity offers IM residents effective initial nutrition training for patients at risk for cardiovascular disease and is practical to implement as part of an ambulatory curriculum.

Keywords
Preventive Medicine, Nutrition, Cardiovascular Disease, Counseling, Primary Care, Cardiovascular Medicine, Complementary/Alternative Medicine, Family Medicine, Internal Medicine, Case-Based Learning, Editor's Choice

Educational Objectives

By the end of this activity, learners will be able to:

1. Improve their own attitudes regarding the central role of the primary care provider in nutrition counseling for patients with diet-related cardiovascular risk factors.
2. Take a focused dietary history.
3. Identify evidence-based techniques for dietary management of hypertension, hyperlipidemia, overweight, and obesity.
4. Initiate personalized nutrition counseling in appropriate patients using behavioral change assessment.
5. Refer appropriate patients to a health care provider specializing in nutrition counseling.

Citation:
Jain S, Feldman R, Althouse AD, Spagnoletti C, Proksell S. A nutrition counseling curriculum to address cardiovascular risk reduction for internal medicine residents. *MedEdPORTAL*. 2020;16:11027.
https://doi.org/10.15766/mep_2374-8265.11027

Introduction

Dietary intervention is a principal component in the management of cardiovascular disease and its diet-related risk factors, including hypertension, hyperlipidemia, diabetes, and obesity.[1] Dieticians are not a ubiquitous resource, however; patients' access to dieticians depends on their diagnoses, access to care, and insurance. Thus, primary care providers play a critical role in providing dietary management to reduce cardiovascular risk. Numerous barriers prevent physicians from providing adequate nutrition counseling, such as lack of time, uncertainty of effectiveness of counseling, inadequate skills in providing counseling, lack of a systematic approach, and need for collaboration with other health care professionals.[1] Because of these barriers, rates of dietary counseling by physicians are low, even for patients with cardiovascular disease, diabetes, or

Copyright © 2020 Jain et al. This is an open-access publication distributed under the terms of the Creative Commons Attribution license.

hyperlipidemia, with rates of counseling ranging from 10% to 40% of primary care visits.[2,3]

Improving nutrition education for trainees in primary care specialties is a critical component in addressing this deficiency in care. Residents in primary care specialties should be able to identify patients at risk for malnutrition, initiate nutrition counseling in appropriate patients, and refer to other health care professionals when indicated. However, only a minority of physicians believe they have been adequately trained in nutrition counseling.[3,4] Review of the literature on nutrition education reveals that most educational interventions are targeted towards medical students, not residents.[5,6] In a survey provided to program directors across multiple residency programs, 80% of which were primary care–related specialties, only 26% of programs had a formal curriculum for nutrition, despite 72% of program directors believing a nutrition course should be required for their residents.[7] There is no formal system for nutrition education in internal medicine residency; the Accreditation Council for Graduate Medical Education guidelines do not contain any nutrition-based competencies and the board exam does not test competency in nutrition counseling.[1]

A national survey of internal medicine residents and program directors indicated that both the amount and number of instructional methods on nutrition education predicted frequency of residents' dietary counseling practices.[3] One randomized multicenter study performed at resident continuity clinics showed that an educational program on dietary counseling increased the percentage of physicians who felt confident in providing effective dietary counseling from 26% to 67%-78%.[8] However, most interventions focused solely on self-reported changes in attitude and skills as outcome targets and did not objectively assess improvement in trainee counseling skills.[8,9]

We developed and implemented a nutrition curriculum to improve the ambulatory nutrition counseling provided by our program's internal medicine residents for patients with cardiovascular risk factors. We chose preclinic conference sessions for our venue as these sessions were already built into the ambulatory curriculum, were widely utilized throughout internal medicine residency programs nationwide, and allowed for enhanced interaction and discussion due to the small-group format. We structured each session around patient cases because of the potential for case-based learning to provide relevance to the adult learner and induce changes in behavior based on knowledge learned.[10] We incorporated several evidence-based learning strategies[11] into our curriculum: We provided assessment questions at the end of each session for retrieval practice, used a worksheet format to encourage note-taking, and spread out the curriculum over two sessions held a week apart for spaced learning. We studied the effect of our intervention on resident knowledge, behavior, and skills via both self-report in surveys and outcomes collected from the electronic health records (EHRs) of the patients in our resident clinics.

Methods

Our curricular intervention was approved by the University of Pittsburgh Medical Center Quality Improvement Review Committee (Project ID 1826) in August 2018 and did not require additional institutional review board oversight. We developed two case-based learning activities for internal medicine residents to be given during two preclinic conference sessions. No prerequisite knowledge was needed by learners. Facilitators were not required to have prerequisite knowledge to lead the sessions, although some practical experience providing nutrition counseling to patients in a primary care setting and using motivational interviewing techniques, as well as familiarity with the transtheoretical stages of change model, would certainly have enhanced discussion. Each session lasted approximately 45 minutes and utilized patient cases to outline major learning points. In the first session, we discussed nonpharmacologic management of patients with hypertension, hyperlipidemia, and body mass index (BMI) ≥ 25 and reviewed evidence for relevant dietary patterns (Appendices A and B). In the second session, we provided resources for dietary referrals available in our health system and illustrated examples of appropriate provider responses to use when counseling based on the stage of behavioral change of the patient. We also provided information on questions frequently asked by patients regarding current diet trends (Appendices C and D). After completion of the second session, we provided a take-home handout reviewing key learning points (Appendix E). Prior to delivery, Appendices A-E were reviewed with a registered dietician at our institution who had extensive experience working in primary care and cardiology clinics, as well as with several academic general internists with training in medical education.

Our curriculum was delivered to internal medicine residents caring for patients at our two university clinic sites, sites A and B. Based on demographics from 2017 to 2019, about twice as many patients were seen at site A compared to site B. About half of patients seen at both resident clinics were female, about half of patients were White, and approximately one-third were Black. Patients seen at site B were slightly older. Approximately one-third of patients at both sites were obese. Percentages of patients with coronary artery disease, hypertension, and diabetes

Copyright © 2020 Jain et al. This is an open-access publication distributed under the terms of the Creative Commons Attribution license.

were similar at both sites. A higher percentage of patients at site A used medical assistance or had no insurance. Resources available for nutrition counseling were similar at both sites.

We evaluated our intervention using surveys and data obtained from EHRs. A series of electronic surveys were sent to participants prior to curriculum delivery, immediately after, and 2 months after (Appendix F). Participants were given time during the first session to complete the presurvey and during the second session to complete the initial postsurvey. The delayed postsurvey was sent electronically 2 months later. The surveys were created with Qualtrics software and were piloted in an iterative process with internists with training in medical education. Survey questions addressed knowledge, attitudes regarding the role of the primary care provider in dietary counseling for patients with elevated cardiovascular risk, and frequency of performing tasks related to nutrition counseling for patients with obesity, hypertension, and hyperlipidemia. Questions assessing medical knowledge were adapted from online modules.[12,13]

We also assessed outcomes via the EHRs of patients in our resident clinics who had a BMI ≥ 25 kg/m[2], a face-to-face encounter with a resident provider within the time period specified, and inclusion of overweight, obesity, hypertension, or hyperlipidemia in the encounter diagnosis. We assessed the frequency of documentation of the following factors in the 2 months prior and 2 months after delivery of our curriculum: obesity or overweight in the problem list or encounter diagnosis, waist circumference, goals made or altered during the visit, referrals placed for nutrition-related counseling (to a dietician, nurse educator, or health coach), and nutrition counseling provided during the visit by the resident primary care provider. Documentation of nutrition counseling was assessed by chart review done by the primary author. Demographic data extracted from the EHR included patient age, gender, and BMI, as well as the postgraduate (PGY) level of the resident provider.

Statistical Methods

Descriptive statistics are presented as *M* (*SD*) for continuous variables and *n* (%) for categorical variables. The number and frequency of responses on pre- versus postsurveys were compared using McNemar's test. For the outcomes analysis, multivariable logistic regression was used to estimate whether patients seen after the intervention versus before the intervention were more likely to have documented nutrition counseling or referrals to nutrition counseling when adjusting for number of diagnosis, degree of obesity, and clinical site. A random effect was included in each model to account for clustering by resident (e.g., dependency of participants seen by the same resident).

Results

Seventy-six internal medicine residents ranging from PGY 1 to PGY 3 completed the curriculum. Fifty-four residents completed the initial survey. Demographics of respondents completing the initial survey ($n = 54$) are included in Table 1: Forty-three percent were female, mean age was 28, and 46% of residents were at a PGY 1 training level. Most survey respondents (77%) noted that they had received no formalized training in nutrition prior to residency. The most common intended career paths included gastroenterology (20%), cardiology (19%), and general medicine (13%). There were 25 paired responses for the pre- and immediate postsurveys, 12 paired responses for the pre- and delayed postsurveys, and 11 paired responses for all three surveys.

Among those who completed a pre- and postsurvey, resident attitudes towards the primary care provider's role in nutrition counseling for patients with obesity, hypertension, or hyperlipidemia remained mostly unchanged after receiving the curriculum. Both prior to and after receiving the curriculum, most survey respondents believed it was the responsibility of the primary care provider to both identify appropriate patients for nutrition counseling and perform the counseling themselves ($n = 25$ paired responses).

Table 1. Demographics of Respondents From the Presurvey ($n = 54$)

Variable	No. (%)	*M* (*SD*)
Gender		
Female	23 (43)	
Male	31 (57)	
Age (years)		28.0 (1.9)
Postgraduate year		
1	25 (46)	
2	17 (32)	
3	12 (22)	
Continuity clinic site		
A	30 (56)	
B	24 (44)	
Nutrition training prior to residency		
A dedicated nutrition course in medical school	12 (23)	
Dedicated nutrition training outside of medical school	0 (0)	
No formalized training in nutrition prior to residency	41 (77)	
Intended career choice		
Allergy/immunology	2 (4)	
Cardiology	10 (19)	
Endocrinology	1 (2)	
Gastroenterology	11 (20)	
General medicine	7 (13)	
Geriatrics or palliative care	1 (2)	
Hematology/oncology	4 (7)	
Infectious disease	3 (6)	
Nephrology	2 (4)	
Pulmonology/critical care	5 (9)	
Rheumatology	2 (4)	
Undecided	6 (11)	

Abbreviations: *M,* mean; *SD,* standard deviation.

Copyright © 2020 Jain et al. This is an open-access publication distributed under the terms of the Creative Commons Attribution license.

Resident knowledge of nutrition counseling was assessed by multiple-choice questions in the surveys. Aggregate score of the six knowledge questions significantly improved from 67% to 85% in the immediate postsurvey compared to the presurvey (Figure 1; $n = 24$ paired responses, $p = .004$). When comparing those who completed all three surveys ($n = 11$ paired responses), aggregate score for the knowledge questions was 65% in the precurriculum survey, 80% in the immediate postcurriculum survey, and 74% in the delayed postcurriculum survey.

Resident skills related to nutrition counseling were partly assessed by self-report in the presurvey and delayed postsurvey (Table 2 ; $n = 12$ paired responses). For patients with obesity, 11 residents (92%) reported providing nutrition counseling to appropriate patients always or most of the time in the delayed postcurriculum survey, an increase from seven residents (58%) at baseline. For patients with hypertension, seven residents (58%) reported obtaining a dietary history always or most of the time in the delayed postsurvey, an increase from four residents in the presurvey (33%). Furthermore, report of providing nutrition counseling in appropriate patients always or most of the time increased from six (50%) to 10 residents (83%). For patients with hyperlipidemia, 10 residents (83%) reported providing nutrition counseling always or most of the time in the delayed postsurvey, compared to five residents (42%) at baseline. In both surveys, for patients with obesity, no participants reported measuring waist circumference for additional risk stratification. Additionally, in both surveys, for patients with obesity, hypertension, or hyperlipidemia, zero to one participants reported using a dietary assessment tool when taking a dietary history.

We also examined data from the EHRs of patients with overweight or obesity, hypertension, and/or hyperlipidemia who were seen in a face-to-face encounter with residents 2 months prior ($n = 503$) and 2 months after ($n = 473$) the implementation of the curriculum (Table 3). The demographics of the patients in these two time periods were similar: Mean age was 56, 51% of patients were female, and mean BMI was 34. The distribution of PGY level for resident providers was also similar; on average, 26% of providers were PGY 1, 41% were PGY 2, and 33% were PGY 3. More patients were seen at clinic site A (64% of encounters) compared to clinic site B (36% of encounters).

Rates of documented nutrition counseling increased from 35% to 41% in the postintervention EHR data set compared to preintervention rates (odds ratio [OR], 1.27; 95% CI, 0.967-1.667; $p = .085$), although we cannot conclude that the intervention alone was responsible for this change. This relationship remained consistent when adjusting for number of diagnoses, BMI category, or clinical site. Referrals for nutrition counseling were placed in only 8% of encounters, and frequency of referrals remained unchanged pre- and postintervention (OR, 1.01; 95% CI, 0.632-1.619; $p = .963$); rates remained unchanged when adjusting for number of diagnoses, BMI category, and resident provider. Notably, the odds of placing referrals for nutrition counseling were significantly lower at site B compared to site A (OR, 0.57; 95% CI, 0.334-0.982; $p = .043$). Both referrals for nutrition counseling and documented nutrition counseling provided by the resident primary care provider were significantly more likely during encounters when the patient had more than one of the included diagnoses (overweight, obesity,

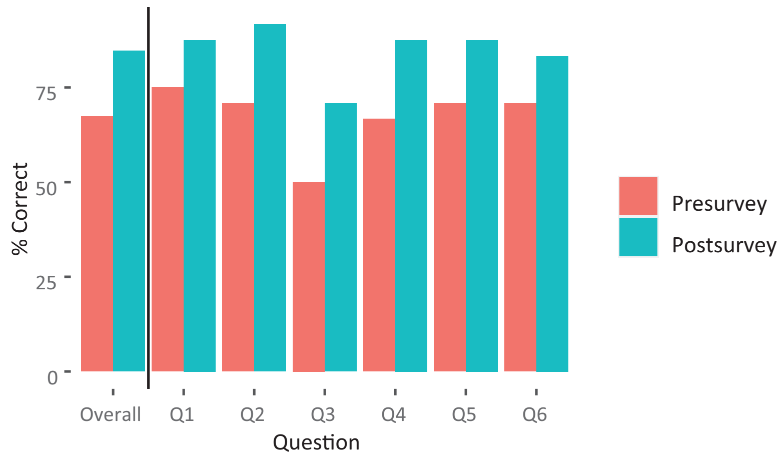

Figure 1. Percent correct scores for the six knowledge questions in the pre- and immediate postsurveys ($n = 24$ paired responses). When comparing mean difference in overall score, $p = .004$. Abbreviation: Q, question.

Copyright © 2020 Jain et al. This is an open-access publication distributed under the terms of the Creative Commons Attribution license.

Table 2. Comparison of Responses Regarding Frequency of Counseling-Related Behavior From the Precurriculum and Delayed Postcurriculum Surveys ($n = 12$)[a]

Condition	Statement and Responses	No. (%) Pre	No. (%) Delayed
Obesity			
	I list weight as a separate problem or diagnosis in the electronic health record.		
	Always	1 (8)	2 (17)
	Most of the time	5 (42)	4 (33)
	Never	0 (0)	2 (17)
	Some of the time	6 (50)	4 (33)
	I measure waist circumference for additional risk stratification.		
	Never	11 (92)	11 (92)
	Some of the time	1 (8)	1 (8)
	I take a dietary history when addressing obesity.		
	Always	4 (33)	5 (42)
	Most of the time	3 (25)	4 (33)
	Never	3 (25)	1 (8)
	Some of the time	2 (17)	2 (17)
	I use a dietary assessment tool when taking a dietary history.		
	Always	4 (33)	5 (42)
	Most of the time	3 (25)	4 (33)
	Never	3 (25)	1 (8)
	Some of the time	2 (17)	2 (17)
	I provide nutrition counseling to patients for whom I feel it is appropriate.		
	Always	4 (33)	7 (58)
	Most of the time	3 (25)	4 (33)
	Never	1 (8)	0 (0)
	Some of the time	4 (33)	1 (8)
	I refer appropriate patients for nutrition counseling.		
	Always	0 (0)	4 (33)
	Most of the time	5 (42)	3 (25)
	Never	0 (0)	1 (8)
	Some of the time	7 (58)	4 (33)
Hypertension			
	I take a dietary history when addressing hypertension.		
	Always	1 (8)	3 (25)
	Most of the time	3 (25)	4 (33)
	Never	1 (8)	1 (8)
	Some of the time	7 (58)	4 (33)
	I use a dietary assessment tool when taking a dietary history.		
	Never	11 (92)	8 (67)
	Some of the time	1 (8)	4 (33)
	I provide nutrition counseling to patients for whom I feel it is appropriate.		
	Always	3 (25)	6 (50)
	Most of the time	3 (25)	4 (33)
	Never	1 (8)	0 (0)
	Some of the time	5 (42)	2 (17)
	I refer appropriate patients for nutrition counseling.		
	Always	1 (8)	3 (25)
	Most of the time	4 (33)	3 (25)
	Never	2 (17)	4 (33)
	Some of the time	5 (42)	2 (17)
Hyperlipidemia			
	I take a dietary history when addressing hyperlipidemia.		
	Always	3 (25)	0 (0)
	Most of the time	3 (25)	7 (58)
	Never	2 (17)	2 (17)
	Some of the time	4 (33)	3 (25)
	I use a dietary assessment tool when taking a dietary history.		
	Always	1 (8)	0 (0)
	Most of the time	0 (0)	1 (8)
	Never	10 (83)	7 (58)
	Some of the time	1 (8)	4 (33)
	I provide nutrition counseling to patients for whom I feel it is appropriate.		
	Always	2 (17)	3 (25)
	Most of the time	3 (25)	7 (58)

(*Table Continues*)

Copyright © 2020 Jain et al. This is an open-access publication distributed under the terms of the Creative Commons Attribution license.

Table 2. (*Continued*)

Condition	Statement and Responses	No. (%)	
		Pre	Delayed
	Never	2 (17)	0 (0)
	Some of the time	5 (42)	2 (17)
Hyperlipidemia (cont.)	I refer appropriate patients for nutrition counseling.		
	Always	0 (0)	2 (17)
	Most of the time	4 (33)	5 (42)
	Never	2 (17)	3 (25)
	Some of the time	6 (50)	2 (17)

[a]The sample size was too small for meaningful statistical analysis; thus, *p* values are not shown. The best answer for each question was "always" or "most of the time."

hypertension, or hyperlipidemia) or had a BMI in a higher weight class.

Discussion

The dearth of nutrition education for resident physicians in primary care fields is well reported.[1-4] We delivered a curriculum to help improve the nutrition counseling skills of internal medicine residents in our program in the ambulatory setting.

We learned several valuable lessons while implementing our educational sessions. While the second session was well timed, the first session felt rushed for the 45 minutes allotted to it. To thoroughly review the evidence behind the guideline-based recommendations discussed in Appendix A, the first session could be split into two separate sessions. For instance, session one could review Appendix A, question 1: risks for cardiovascular disease, recommendations for weight loss, and

Table 3. Patient and Provider Data Obtained From Electronic Health Records of Patients Seen in Two Resident Clinics, Comparing Precurriculum and Postcurriculum Data

Measure and Category	Pre (n = 503)		Post (n = 473)		
	No. (%)	*M* (*SD*)	No. (%)	*M* (*SD*)	*p*
Patient characteristics					
Gender					
Female	268 (53)		230 (49)		.146
Male	235 (47)		243 (51)		
Age on visit date (years)		56.4 (13.9)		56.2 (13.7)	.853
BMI (kg/m^2)		33.8 (6.8)		34.2 (7.4)	.426
BMI class[a]					
Overweight	178 (35)		160 (34)		.635
Obesity class I	152 (30)		136 (29)		
Obesity class II	81 (16)		91 (19)		
Obesity class III	92 (18)		86 (18)		
Clinic site					
A	344 (68)		283 (60)		.005
B	159 (32)		190 (40)		
Number diagnoses		1.7 (0.8)		1.8 (0.8)	.617
ICD-10 code for overweight and obesity present					
No	325 (65)		297 (63)		.554
Yes	178 (35)		176 (37)		
Provider characteristics					
Postgraduate year					
1	144 (29)		111 (24)		.113
2	192 (38)		207 (44)		
3	167 (33)		155 (33)		
Referral for nutrition counseling provided					
No	464 (92)		436 (92)		.968
Yes	39 (8)		37 (8)		
Nutrition counseling documented					
No	325 (65)		279 (59)		.07
Yes	178 (35)		194 (41)		

Abbreviations: BMI, body mass index; ICD-10, International Classification of Diseases, 10th revision; *M*, mean; *SD*, standard deviation.
[a]BMI class: overweight = BMI 25.0-29.9 kg/m^2, obesity class I = BMI 30.0-34.9 kg/m^2, obesity class II = BMI 35.0-39.9 kg/m^2, obesity class III = BMI 40.0+ kg/m^2.

Copyright © 2020 Jain et al. This is an open-access publication distributed under the terms of the Creative Commons Attribution license.

evidence for Mediterranean and plant-based diets. Session two could review Appendix A, questions 2 and 3: evidence-based dietary recommendations for patients with hypertension and hyperlipidemia. To provide even greater depth, sessions could be cofacilitated by a dietician and a physician preceptor. Additionally, in our second session, when given sample patient statements to respond to, residents may have more successfully improved counseling techniques if they paired up with each other and practiced role-playing as patient and provider.

Regarding our first aim, improving resident attitudes towards nutrition counseling in the ambulatory setting, we found that even before our curriculum, most of our residents believed it was the responsibility of the primary care provider to both identify appropriate patients and provide nutrition counseling to patients with obesity, hypertension, or hyperlipidemia; these beliefs remained unchanged after curriculum delivery. Similarly, a survey of 61 internal medicine interns at one program indicated positive attitudes about nutrition assessment and counseling, regardless of prior training in nutrition and despite reporting inadequate proficiency in providing counseling.[14]

As most trainees and physicians feel inadequately trained to advise patients on dietary counseling,[4,14] we aimed to improve residents' knowledge of evidence-based dietary management techniques to reduce cardiovascular risk. Our results demonstrated that after delivery of our curriculum, trainee knowledge of the nutrition interventions available for reducing cardiovascular risk improved. Participants retained this knowledge as well at 2 months, although our evidence is limited by the low response rates of the delayed postsurvey and the low number of knowledge questions in each survey (six). Still, this is a promising first step to establishing formalized nutrition education for residents in primary care fields. Several teaching methods described in the literature could be used to build a comprehensive nutrition curriculum for residents.[3-6] Sessions could be cofacilitated by a dietician to expand the depth of knowledge provided and to encourage interprofessional education. Additionally, workshops with teaching kitchens could be integrated into the ambulatory curriculum to provide residents with improved competency in culinary education.

In addition to improving resident knowledge, we also aimed to teach residents to initiate personalized nutrition counseling. We provided numerous sample patient statements in our curricular activity for residents to practice appropriate individualized responses, incorporating behavioral change assessment into their counseling. According to our postsurvey data, more residents reported providing nutrition counseling for appropriate patients in their clinic. Furthermore, we observed a trend toward increased EHR documentation of nutrition counseling provided by residents after curriculum delivery, although we cannot conclude that the intervention alone was responsible for this increase. Notably, we used documentation of counseling as a surrogate for the actual counseling provided by the resident primary care provider, and thus, we do not know the quality or extent of the counseling provided in each encounter. In addition, there may have been encounters where lifestyle counseling was provided but not documented. Additional teaching tools are necessary to train residents to provide nutrition counseling, as both the amount and number of instructional methods in nutrition education predict frequency of residents' dietary counseling practices.[3] For example, trainees could partner up to assess each other's diets and readiness for change, practicing counseling techniques with each other. Additionally, trainees could practice counseling in one-on-one or group sessions with standardized patients.

Another potential instrument to improve personalized nutrition counseling by trainees is the implementation of dietary assessment tools. Participants in our surveys reported zero to minimal use of dietary assessment tools as part of history taking for patients with hypertension, hyperlipidemia, or obesity. In our curricular activity, we provided an example of a dietary assessment tool, Starting the Conversation, a brief screening tool validated in patients with type 2 diabetes and available in the public domain.[2] Perhaps use of such tools would increase if incorporated into intake forms for new patients and annual wellness visits.

Our final objective was to teach residents to refer appropriate patients to health care providers specializing in nutrition counseling. We explained in the second session the relevant resources available at sites A and B (which included dieticians, nurse educators, and health coaches) and how to place orders for these referrals. Given that this was relatively simple to do, it was surprising that rates of referrals were so low both prior to and after curriculum delivery. However, we do not know how often trainees were offering these services, only how frequently the services were ordered. One explanation for low rates of referrals could be that many patients in the resident clinics had significant social determinants of health and barriers to accessing care; perhaps their readiness to change was poor and it was not the time to place a referral for dietary counseling, or the patient declined the referral. Notably, odds of placing referrals for nutrition counseling were significantly lower at site B compared to site A. Perhaps additional site-specific reminders on available

Copyright © 2020 Jain et al. This is an open-access publication distributed under the terms of the Creative Commons Attribution license.

resources are needed. Additionally, interprofessional teaching sessions led by a registered dietician could be incorporated into the ambulatory curriculum.[15] Clinics that offer alternative delivery models such as group medical visits, where patients elect to attend group sessions with a dietician, would likely also help address the lack of nutrition counseling facing many primary care patients.

As our activity can be delivered over two to three 45-minute small-group sessions, we feel it is practical to implement as part of a residency's busy ambulatory curriculum. As we have discussed, however, greater depth may be achieved if the sessions are instead cofacilitated by a physician and a dietician. Limitations of our study include the low response rates of our immediate and delayed postcurriculum surveys, limiting our ability to detect significant changes in participant behavior. In addition, we did not discriminate between resident providers who did and did not complete the curriculum when evaluating EHR data. Furthermore, our experience of curricular implementation was limited to one academic internal medicine residency program. Our educational activity provides effective initial nutrition education to improve residents' lifestyle counseling skills for patients at risk for cardiovascular disease. Although it is not a comprehensive curriculum for ambulatory nutrition education, we feel our curriculum would be a valuable component of a residency program's efforts to build trainees' skills in nutrition counseling.

Appendices

A. Session 1 Preceptor Handout.docx

B. Session 1 Resident Handout.docx

C. Session 2 Preceptor Handout.docx

D. Session 2 Resident Handout.docx

E. Take-Home Handout.docx

F. Pre-and Postsurvey.docx

All appendices are peer reviewed as integral parts of the Original Publication.

Seema Jain, MD: Assistant Instructor, Division of General Internal Medicine, University of Texas Southwestern Medical Center

Robert Feldman, MS: Data Analyst, Center for Research on Health Care (CRHC) Data Center, University of Pittsburgh

Andrew D. Althouse, PhD: Assistant Professor, Division of General Internal Medicine, University of Pittsburgh School of Medicine

Carla Spagnoletti, MD: Professor, Division of General Internal Medicine, University of Pittsburgh School of Medicine

Siobhan Proksell, MD: Assistant Professor, Division of Gastroenterology, Hepatology, and Nutrition, University of Pittsburgh School of Medicine

Disclosures

None to report.

Funding/Support

None to report.

Ethical Approval

Reported as not applicable.

References

1. Lenders CM, Deen DD, Bistrian B, et al. Residency and specialties training in nutrition: a call for action. *Am J Clin Nutr*. 2014;99(5):1174S-1183S. https://doi.org/10.3945/ajcn.113.073528
2. Kahan S, Manson JE. Nutrition counseling in clinical practice: how clinicians can do better. *JAMA*. 2017;318(12):1101-1102. https://doi.org/10.1001/jama.2017.10434
3. Khandelwal S, Zemore SE, Hemmerling A. Nutrition education in internal medicine residency programs and predictors of residents' dietary counseling practices. *J Med Educ Curric Dev*. 2018;5: 2382120518763360. https://doi.org/10.1177/2382120518763360
4. Eisenberg DM, Burgess JD. Nutrition education in an era of global obesity and diabetes: thinking outside the box. *Acad Med*. 2015;90(7):854-860. https://doi.org/10.1097/acm.0000000000000682
5. Wilechansky R, Burgermaster M, Jones D, Seres D. Obesity, diet, and exercise education for the primary care clerkship using an Articulate Storyline 2 e-learning module. *MedEdPORTAL*. 2016; 12:10497. https://doi.org/10.15766/mep_2374-8265.10497
6. Miller DP, Crandall SJ, Davis S, et al. NEW LifeStyle: a downloadable teaching and learning program for nutrition, exercise, and weight management. *MedEdPORTAL*. 2012;8:9256. https://doi.org/10.15766/mep_2374-8265.9256
7. Daley BJ, Cherry-Bukowiec J, Van Way CW III, et al; A.S.P.E.N. Task Force on Postgraduate Medical Education. Current status of nutrition training in graduate medical education from a survey of residency program directors: a formal nutrition education course is necessary. *JPEN J Parenter Enteral Nutr*. 2016;40(1):95-99. https://doi.org/10.1177/0148607115571155
8. Evans AT, Rogers LQ, Peden JG Jr, et al; CADRE Study Group. Teaching dietary counseling skills to residents: patient and physician outcomes. *Am J Prev Med*. 1996;12(4):259-265. https://doi.org/10.1016/S0749-3797(18)30322-2

Copyright © 2020 Jain et al. This is an open-access publication distributed under the terms of the Creative Commons Attribution license.

9. Lang RD, Jennings MC, Lam C, Yeh HC, Zhu C, Kumra T. Community culinary workshops as a nutrition curriculum in a preventive medicine residency program. *MedEdPORTAL*. 2019;15:10859. https://doi.org/10.15766/mep_2374-8265.10859
10. McLean SF. Case-based learning and its application in medical and health-care fields: a review of worldwide literature. *J Med Educ Curric Dev*. 2016;3:39-49. https://doi.org/10.4137/jmecd.s20377
11. Brown PC, Roediger HL III, McDaniel MA. *Make It Stick: The Science of Successful Learning*. Harvard University Press; 2014.
12. Adams KM, Kohlmeier M, Briggs Early K. Effective weight loss strategies for adults. *Nutrition in Medicine*. 2013. http://www.nutritioninmedicine.org/portal/
13. Gudzune K, Chaudry ZW, Clark JM. Obesity and overweight: diagnosis and management. *Physician Education and Assessment Center: Internal Medicine Curriculum*. 2018. https://ilc.peaconline.org/
14. Vetter ML, Herring SJ, Sood M, Shah NR, Kalet AL. What do resident physicians know about nutrition? An evaluation of attitudes, self-perceived proficiency and knowledge. *J Am Coll Nutr*. 2008;27(2):287-298. https://doi.org/10.1080/07315724.2008.10719702
15. Hark LA, Deen D. Position of the Academy of Nutrition and Dietetics: interprofessional education in nutrition as an essential component of medical education. *J Acad Nutr Diet*. 2017;117(7): 1104-1113. https://doi.org/10.1016/j.jand.2017.04.019

Received: March 22, 2020
Accepted: July 7, 2020
Published: November 11, 2020

Copyright © 2020 Jain et al. This is an open-access publication distributed under the terms of the Creative Commons Attribution license.
